# High-temperature requirement serine protease A2 inhibitor UCF-101 ameliorates damaged neurons in traumatic brain-injured rats by the AMPK/NF-κB pathway

**DOI:** 10.1515/biol-2022-0971

**Published:** 2025-03-06

**Authors:** Tian-Wen Qiu, Zhan Jin, Zhi-Zhan Fu, Xin-Jiang Yan, Cheng-Peng Zhan, Hui-Wen Zheng, Mei-Ying Li, Guo-Feng Yu

**Affiliations:** Postgraduate Cultivation Base, The Quzhou Affiliated Hospital of Wenzhou Medical University, Quzhou, 324000, Zhejiang, China; School of Medicine, Quzhou College of Technology, Quzhou, 324000, Zhejiang, China; Department of Neurosurgery, Quzhou People’s Hospital, No. 100, Minjiang Avenue, High-speed Rail New City, Quzhou, 324000, Zhejiang, China

**Keywords:** UCF-101, traumatic brain injury, AMPK/NF-κB, inflammation factor

## Abstract

Traumatic brain injury (TBI) leads to permanent damage, including neurological deficits, cognitive deficits, and cerebral edema. The specific inhibitor of serine protease Omi/high-temperature requirement A2 (Omi/HtrA2), UCF-101, exerts neuroprotective effects, but its role in TBI remains unclear. Eighty-four male Sprague Dawley rats were randomized to control, TBI, UCF-101 of low dose (1.5 μmol/kg), middle dose (3.0 μmol/kg), and high dose (6.0 μmol/kg), Compound C (AMPK inhibitor, 20 mg/kg), and high dose + Compound C groups. TBI rat modeling was operated by the controlled cortical impact method. Modified neurological severity score (mNSS) cognitive function, cerebral edema index, hematoxylin-eosin staining, TUNEL staining for apoptosis, ethidium bromide staining for blood–brain barrier (BBB) permeability, enzyme-linked immunosorbent assay for inflammation response, and Western blot analysis were performed. In TBI rats, UCF-101 caused decreased mNSS score, brain edema, neuronal apoptosis, as well as P-NF-κBp65/NF-κBp65, tumor necrosis factor-α, interleukin (IL)-1β, and IL-8 expression, while P-AMPK/AMPK, zonula occludens protein, Occludin, and Claudin-5 expression increased, accompanied with up-regulated cognitive function. Moreover, Compound C further exacerbated brain tissue lesions, neuronal damage, inflammation response, and neuronal apoptosis, while high-dose UCF-101 offset its effect. UCF-101 may inhibit apoptosis and BBB permeability to exert neuroprotective effects in TBI rats by regulating the AMPK/NF-κB pathway, advancing UCF-101 clinical applications for TBI treatment.

## 1 Introduction

Traumatic brain injury (TBI), which occurs easily during strenuous exercise or traffic accidents, is one of the most common critical illnesses in clinical practice [1]. There are approximately 55 million people worldwide suffering from this disease [2]. The probably best characteristic of TBI is heterogeneity [3]. Although it is an acute acquired injury to the brain due to an external mechanical impact, it may develop with unique permanent impairments over time, including neurological deficits, cognitive deficits, sleep disturbances, neurodegenerative disorder, and metabolic disorders, that is, primary and secondary injury two stages [4,5,6]. The serious secondary cerebral edema caused by TBI involves severe cytotoxicity and intracranial hypertension, leading to life-threatening effects [7,8]. Therefore, developing feasible and personalized treatment for TBI is imperative.

The pathogenesis of TBI is complex, primarily focusing on neuroinflammatory responses, blood–brain barrier (BBB) destruction, oxidative damage, and mitochondrial dysfunction. However, the exact mechanism of TBI remains unclear [8]. COG1410 was found to play a role in improving TBI by alleviating BBB destruction and brain edema, down-regulating tumor necrosis factor-α (TNF-α), interleukin (IL)-1β, Bcl2-associated x (Bax), and cleaved caspase-3, as well as inhibiting neural apoptosis to reduce neural damage and improve cognitive function [9]. Abbasloo et al. [10] reported that carvacrol was capable of displaying antioxidant activity to inhibit TBI-induced increased BBB permeability, with lowered oxidative stress levels, whereas enhanced zonula occludens protein (ZO-1), Occludin and Claudin-5 expression, promising it to be a potential therapeutic measure for TBI. Therefore, inhibition of inflammatory response and BBB permeability has important regulatory roles in improving TBI.

Omi/high-temperature requirement A2 (Omi/HtrA2), a mitochondrial serine protease has the potential to be a therapeutic target for TBI, and its activity is essential for cell survival [11,12]. The Omi/HtrA2 inhibition led to decreased apoptosis in external injury stimuli [13]. In fact, the activated Omi/HtrA2 protease induced apoptosis in caspase-dependent and caspase-independent manners, producing active cleaved caspase-3 and caspase-9 to participate in neuroapoptosis [14]. Therefore, as a serine protease inhibitor of Omi/HtrA2 [15], UCF-101 may exert ameliorative effects on TBI. In retinal ischemia/reperfusion mice, UCF-101 intervention reduced retinal ganglion cell apoptosis and inhibited the up-regulation of Bax, cleaved caspase-3, and cleaved caspase-9, alleviating retinal inflammation and apoptosis [14]. Furthermore, Wang et al. [16] demonstrated that UCF-101 treatment attenuated brain damage and cognitive deficits to exerted neuroprotective effects in rats with sepsis-associated encephalopathy, mainly reflected in the reduction of caspase-3 and caspase-9 levels, improvement of BBB integrity, increased Occludin, Claudin-5, and ZO-1 expression, as well as suppression of inflammatory responses and oxidative stress. As per these above findings, UCF-101 may exert an ameliorative effect on TBI by inhibiting inflammation, apoptosis, and BBB permeability.

Recognized as an energy-sensing serine/threonine kinase, AMPK plays a nuclear role at the cellular and whole organism level [17]. Moreover, AMPK protects the brain issues by inhibiting neuroinflammation mediated by NF-κB [18]. NF-κB is associated with inflammation processes, which, once activated, further activated TNF-α and IL-6 levels, resulting in neuronal death [19,20]. It was revealed that the AMPK/NF-κB pathway was involved in TBI progression [18,21]. Hesperetin ameliorated TBI by inhibiting microglia-mediated inflammatory responses through activation of AMPK/SIRT1 and inhibition of FoxO1/NF-κB signaling pathway to alleviate neurological impairments, cerebral edema, and neuronal apoptosis in TBI mice, notably, these neuronal protective effects of hesperidin were significantly reversed by AMPK inhibitor Compound C [18]. Bezafibrate was found to alleviate TBI-induced BBB injury by activating the AMPK pathway, with increased ZO-1 expression and improved cognitive function and brain edema [21]. Despite the lack of direct evidence for an association between UCF-101 and the AMPK/NF-κB signaling pathway, UCF-101 inhibited apoptosis in cerebral ischemia/reperfusion by suppressing the p38 MAPK and up-regulating ERK signaling pathway [22]. Meanwhile, AMPK acts as a downstream effector of MAPK signaling and can also inversely regulate the MAPK signaling pathway [23]. More importantly, it was confirmed that inhibition of the MAPK pathway was able to activate the LKB1/AMPK signaling pathway [24]. According to these findings, we further made the hypothesis as below: UCF-101 may inhibit apoptosis and BBB permeability to ameliorate damaged neurons in TBI rats by regulating the AMPK/NF-κB pathway.

Hence, we conducted preliminary exploration by developing TBI rat models to clarify whether UCF-101 may inhibit apoptosis and BBB permeability to exert neuroprotective effects in TBI rats by regulating AMPK/NF-κB pathway, offering new promising targets for TBI treatment and advancing UCF-101 clinical applications for TBI treatment.

## 2 Materials and methods

### 2.1 Animals

Eighty-four specific pathogen-free male Sprague Dawley (SD) rats (250–280 g, 6–8 weeks), obtained from Shanghai Jihui Laboratory Animal Care Co., Ltd, were accommodated at Zhejiang Eyong Pharmaceutical R&D Co., Ltd (No. SYXK (Zhe) 2023-0027). The rearing temperature was 20–26°C, with a relative humidity of 50–60% and a 12-h light–dark cycle. When operating experiments, we followed the regulations of the Institutional Animal Care and Use Committee to alleviate the suffering of rats.


**Ethical approval:** The research related to animal use has been complied with all the relevant national regulations and institutional policies for the care and use of animals and has been approved by the Ethics Committee of Zhejiang Eyong Pharmaceutical Research and Development Center (SYXK (Zhe) 2023-0027).

### 2.2 TBI rat modeling and experiment design

The TBI modeling method was performed with reference to the controlled cortical impact (CCI) method as described previously [9,25]. All rats were fasted for 12 h before operations and were anesthetized with 2% isoflurane and immobilized on a stereotaxic frame. After a brain mid-line skin incision, the periosteum was separated until the parietal bone was exposed. A 5 mm bone window craniotomy was made using a motorized drill located approximately 3.5 mm posterior to bregma and 3.0 mm left of the mid-line. Continuous pressure was then applied by a craniocerebral injury impactor. The impact device applied pressure to the rat brain at 4 m/s impact velocity, 6 mm depth, and 500 ms impact duration time. Thereafter, the bone window was immediately sealed and the scalp was sutured with routine disinfection. Rats were placed on a heated pad to maintain constant normothermia until they recovered. Rats in the control group received the same surgical procedure but no impact injury. Rats exhibited temporary respiratory depression, indicating successful modeling. If there was dural opening, excessive blood loss, and death within 3 h after surgery, these rats were excluded, indicating a modeling failure.

The 84 rats were randomly categorized into 7 groups by random number table method: control, TBI, TBI + low-dose UCF-101 treatment (1.5 μmol/kg, TBI + low), TBI + middle-dose UCF-101 treatment (3.0 μmol/kg, TBI + medium), TBI + high-dose UCF-101 treatment (6.0 μmol/kg, TBI + high), TBI + AMPK inhibitor (20 mg/kg, TBI + Compound C), and TBI + high-dose UCF-101 treatment + AMPK inhibitor (6.0 μmol/kg + 20 mg/kg, TBI + high + Compound C) groups (*n* = 12) [16,22]. UCF-101 (SML1105-5MG, sigma) and AMPK inhibitor, Compound C (BML-317, Selleck), were administered via intraperitoneal injection half an hour before modeling.

### 2.3 Neurological deficit score assessments

The evaluation of Neurological impairment was performed at 24 h after modeling by the modified neurological severity score (mNSS) in terms of motor ability, sensory, balance, reflexes, and abnormal movements, as previously described [26]. The maximum score of 18 indicates severe neurological impairment, with failure on all tasks, a score of 7–12 indicates moderate neurological impairment, and a score of 0–6 indicates mild neurological impairment. To avoid interference of subjective factors, the scores were averaged by the three different investigators blind to group design.

### 2.4 Cognitive function assessments

Following the completion of the mNSS scores, a passive avoidance experiment was conducted to assess the cognitive function of rats. Rats were put into a light box of a passive avoidance recorder, while turning on the recorder, they would enter the dark box without a lighting device because of addiction to darkness. Meanwhile, rats received a foot shock (0.4–1.6 mA), thereby passively obtaining avoidance memory, and were repeated twice to deepen their memory after 2 h. All rats were put into the light box again after 2 h, and the time taken for the first entry to the dark box was recorded as the entry incubation period. The number of electric shocks received by rats entering the dark box was recorded as the number of mistakes. This assessment was also done by three different investigators blind to group design.

## 3 Sample collection

Following completion of cognitive testing, all rats underwent completion of orbital blood sampling before euthanasia by inhalation of compressed CO_2_ gas (30–50% of cage volume/min) in cylinders. At the scene of experiments, rats were disposed of properly only after the animals were confirmed dead. The collection of brain tissues and serum was then completed. Three rats in each group were randomly selected to perform the ethidium bromide (EB) staining, measurement of brain tissue water content, pathology experiments, Western blot assay, and enzyme-linked immunosorbent assay (ELISA), respectively. One part of the collected brain tissues was fixed at 4% polyformaldehyde to prepare paraffin sections for pathology experiments, and the other part of the brain tissues was kept at −80°C.

### 3.1 Hematoxylin-eosin (HE) staining

The prepared paraffin sections were sequentially immersed in xylene and ethanol and then stained in hematoxylin staining solution (H3136, sigma) for 3 min. Following soaked in differentiation solution and bluing buffer, the sections were sequentially immersed in 85% and 95% alcohol and then stained with Eosin staining solution (E4009; Sigma) for 5 min. Dehydration, transparency, and sealing were performed. Then, the observation and image collection were made using a microscope (Nikon Eclipse Ci-L, Nikon). The HE staining was carried out by three different investigators blind to group design.

### 3.2 EB staining

After the passive avoidance experiment, rats were anesthetized and then injected 2 mL/kg of 2% EB solution into the tail vein. The whole brain tissue was taken after 2 h of *in vivo* circulation, rinsed with saline, dried with filter paper, and photographed. The 100 mg brain tissues from the injury area were taken, cut into pieces, placed into 3 mL of formamide solution, homogenized, and incubated in a water bath at 60℃ for 48 h. The centrifugation at 12,000 rpm was made at room temperature for 20 min, and 200 μL of supernatant was added to a 96-well plate. Absorbance measurement was completed at 630 nm wavelength by enzyme labeling instrument. The calculation of EB concentration was carried out under the standard curve, and the EB content of brain tissues was obtained. The calculation formula was as follows: EB content (μg/g) = EB concentration (μg/mL) × formamide volume (mL)/brain tissue mass (g). The higher the EB content, the stronger the permeability of BBB in rats. The EB staining was performed by three different investigators blind to group design.

### 3.3 Brain tissue water content measurement

Brain tissues were collected and the wet weight of brain tissue was weighed. Dry weight was determined after drying at 80℃ for 48 h. The calculation of brain water content was as follows: ([wet weight – dry weight]/wet weight) × 100%.

### 3.4 ELISA

The 200 mg brain tissues taken from the injury area were added into cold PBS solution at a ratio of 1:9, frozen, and centrifuged, and the supernatant was employed to measure TNF-α, IL-1β, and IL-8 levels in accordance with instructions of following kits: TNF-α ELISA kits (Rat, MM-0180R2, Meimian, Jiangsu, China), IL-1β ELISA kits (Rat, MM-0047R2, Meimian, Jiangsu, China), and IL-8 ELISA kits (Rat, ml037351, Meilian, Shanghai, China).

### 3.5 TUNEL staining

The prepared rat brain tissue paraffin sections were taken, and apoptosis was detected by the instructions of the One Step TUNEL Apoptosis Assay Kit (C1090, Beyotime). The sealing with sealing solution containing an anti-fluorescence quench agent was done, and then, collected images were observed under a fluorescence microscope. Under ultraviolet excitation, DAPI-stained cell nuclei exhibited blue color, and positive cell nuclei were stained red. Apoptosis index = positive cell number/total cell number × 100%.

### 3.6 Western blot

Proteins from TBI rats’ brain tissues were extracted, and concentrations were tested using a BCA kit (pc0020; Beyotime). The protein was separated by electrophoresis and then transferred to the polyvinylidene fluoride membrane (10600023; GE Healthcare Life). Following nonspecific antigen block by 5% skim milk powder, primary antibodies (Table 1) were incubated at 4°C overnight. Then, secondary antibodies (Table 1) were incubated at room temperature for 1 h. Protein bands were captured in an ECL luminescence imager (610020-9Q, Clinx). β-Actin was an internal reference, and grayscale values of protein bands were analyzed using ImageJ software.

**Table 1:** Antibody information

Antibody	Company	Article number
ZO-1 antibody	Affinity	AF5145
Occludin antibody	Affinity	DF7504
Claudin-5 antibody	Affinity	AF5216
P-AMPK antibody	Affinity	AF3423
AMPK antibody	Abcam	AB207442
P-NF-κB p65 antibody	Affinity	AF2006
NF-κB p65 antibody	Affinity	AF5006
IL-1β antibody	Proteintech	16806-1-AP
IL-8 antibody	Proteintech	27095-1-AP
TNF-α antibody	ABclonal	A11534
Bax antibody	Affinity	AF0120
Bcl-2 antibody	Affinity	AF6139
Cleaved caspase-3 antibody	Abcam	ab2302
Cleaved caspase-9 antibody	Affinity	AF5240
β-Actin antibody	Proteintech	81115-1-RR
Anti-rabbit IgG, HRP-linked antibody	CST	7074

### 3.7 Statistical analysis

SPSS 20.0 statistical and GraphPad Prism 8 software were applied to conduct data analysis. As measurement data between multiple groups, one-way analysis of variance was for variance analysis when satisfying the criteria for normal distribution and homogeneity of variance. The two-by-two comparisons between groups were made by the Tukey test. In cases where the data were normally distributed but unequal in variance, we used Dunnett’s T3 test or independent samples *t*-test. If it did not fit the normal distribution, Kruskal–Wallis *H*-test was employed. All data were expressed as mean ± standard deviation, with *P* < 0.05 considered statistically significant for all analyses.

## 4 Results

### 4.1 UCF-101 promoted neural function and cognitive function in TBI rats

**Figure 1:**
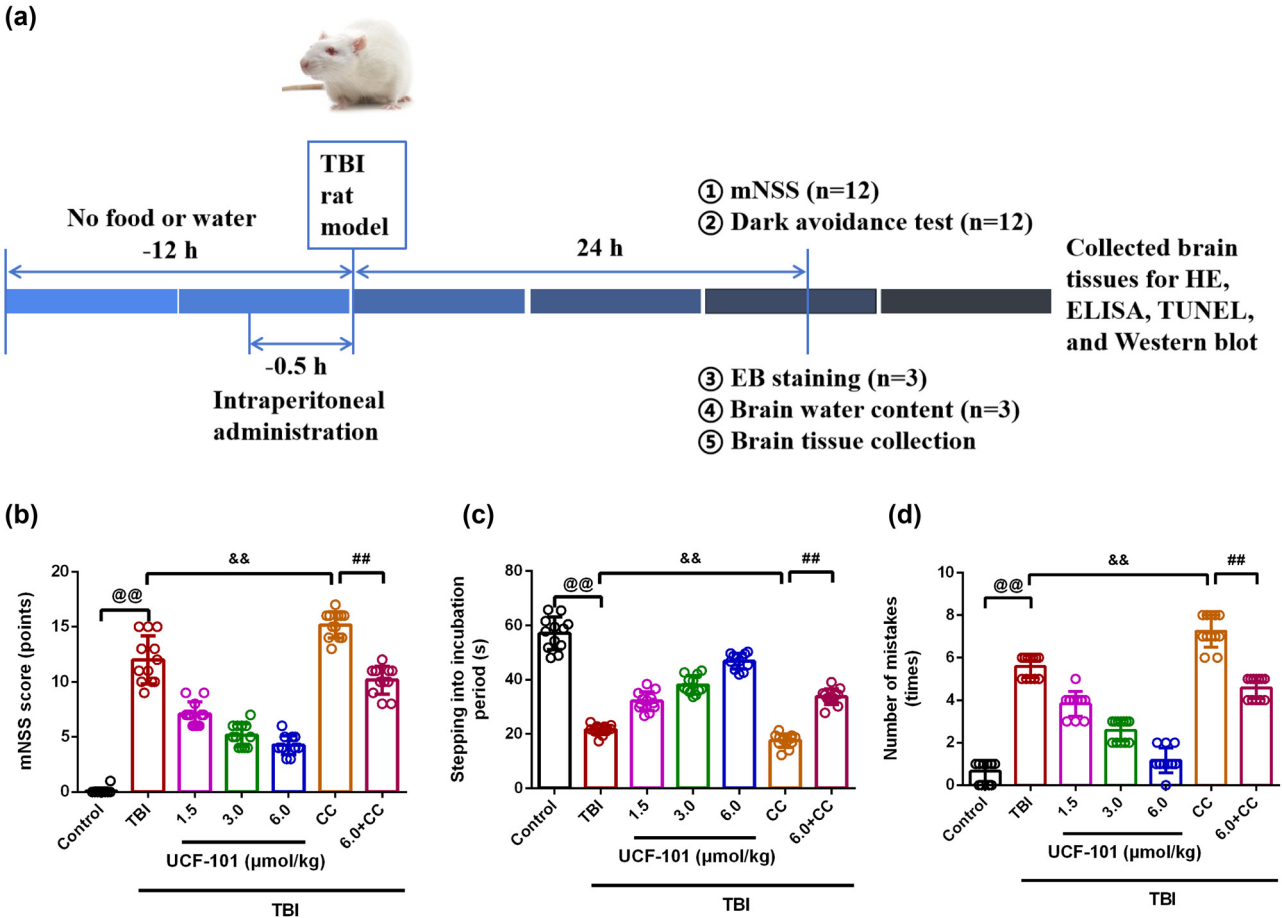
UCF-101 promoted neural function and cognitive function in TBI rats. (a) An experimental flow chart was made. (b) The mNSS score in rats was recorded, *n* = 12. (c) The stepping into the incubation period was determined by passive avoidance experiment, *n* = 12. (d) The number of mistakes was calculated by passive avoidance experiment, *n* = 12; ^@@^ *P* < 0.01 vs Control group, ^&&^ *P* < 0.01 vs TBI group, and ^##^ *P* < 0.01 vs TBI + Compound C group. Note: TBI: traumatic brain injury; CC: Compound C; mNSS: modified neurological severity score; HE: Hematoxylin-eosin.

An experimental flow chart is exhibited in Figure 1a. The mNSS scores were detected to assess the neural function of all rats. There was a significant increase occurred in the mNSS score of TBI rats than in control rats (Figure 1b, *P* = 0.00, 95% confidence interval (CI) 9.55, 14.28). Various doses of UCF-101 treatment for TBI rats decreased the mNSS scores, whereas higher mNSS scores in the TBI + Compound C group than that of the TBI group (Figure 1b, TBI vs TBI + low group: *P* = 0.00, 95% CI 2.45, 7.38; TBI vs TBI + medium group: *P* = 0.00, 95% CI 4.38, 9.29; TBI vs TBI + high group: *P* = 0.00, 95% CI 5.33, 10.17). The TBI + Compound C group exhibited higher mNSS scores compared to the TBI group (*P* = 0.00, 95% CI −5.67, −0.67). Furthermore, in the TBI + high + Compound C group, the addition of high-dose UCF-101 offsets the increase of Compound C on mNSS score in TBI rats (Figure 1b, *P* = 0.00, 95% CI 3.30, 6.70).

In Figure 1c and d, a passive avoidance experiment was performed to assess learning and memory abilities, as well as cognitive abilities in rats. It was found that rats in the TBI group led to shorter stepping into the incubation period and a larger number of mistakes than control rats (*P* = 0.00, stepping into incubation period: 95% CI −42.02, −28.93; number of mistakes: 95% CI 4.22, 5.61). TBI rats exhibited shorter stepping into the incubation period whereas a larger number of mistakes in the TBI + low, TBI + medium, and TBI + high groups (Figure 1c and d, *P* = 0.00, stepping into the incubation period and the number of mistakes: TBI vs TBI + low group: 95% CI −14.45, −6.54 and 95% CI 0.99, 2.51; TBI vs TBI + medium group: 95% CI −20.29, −12.64 and 95% CI 2.29, 3.71; TBI vs TBI + high group: 95% CI −28.56, −21.84 and 95% CI 3.66, 5.17). Moreover, the TBI + Compound C group led to shorter stepping into the incubation period and a larger number of mistakes than those of the TBI group (Figure 1c and d, *P* = 0.00, stepping into incubation period: 95% CI 0.95, 7.04; number of mistakes: 95% CI −2.57, −0.76). On the contrary, TBI rats in the TBI + high + Compound C group demonstrated longer stepping into the incubation period and a smaller number of mistakes than rats in the TBI + Compound C group (Figure 1c and d, *P* = 0.00, stepping into incubation period: 95% CI −19.79, −12.36; number of mistakes: 95% CI 1.76, 3.57). It was evident that Compound C intervention reversed the effects of UCF-101 on the improvement of cognitive function and neurological damage in TBI rats.

### 4.2 UCF-101 treatment attenuated brain injury, BBB permeability, and brain edema in TBI rats

**Figure 2:**
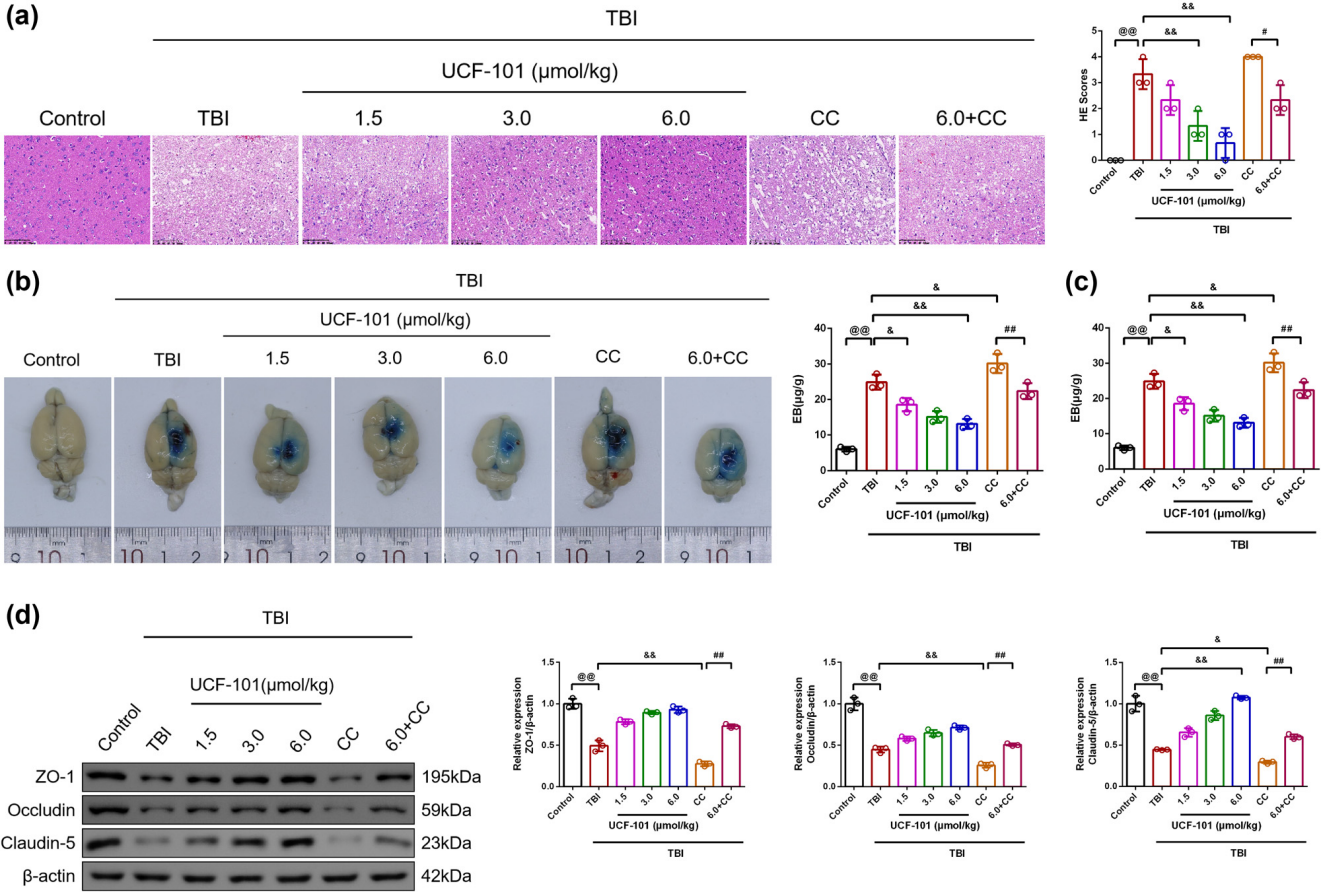
UCF-101 treatment attenuated brain injury, BBB permeability and brain oedema in TBI rats. (a) The pathological changes and HE scores in brain tissues of rats were detected by HE staining (magnification 200×, scale bar: 100 μm), *n* = 3. (b) The EB assay was used to show a visualization of brain tissue from the injury area, *n* = 3. (c) The cerebral edema index was determined by weighing and calculated by the following formula: ([wet weight-dry weight]/wet weight) × 100%, *n* = 3. (d) The protein expression of the ZO-1, Occludin, and Claudin-5 in brain tissues was observed by Western blot, *n* = 3; ^@@^ *P* < 0.01 vs Control group, ^&^ *P* < 0.05 and ^&&^ *P* < 0.01 vs TBI group, and ^#^ *P* < 0.05 and ^##^ *P* < 0.01 vs TBI + Compound C group. Note: BBB: blood–brain barrier; TBI: traumatic brain injury; CC: Compound C; EB: Evans blue; ZO-1: zonula occludens protein; HE: Hematoxylin-eosin.

The pathological changes in rat brain tissues were examined by the HE staining method (Figure 2a). The overall morphology of control rat brain tissue was basically intact, with tightly arranged nerve cells and no obvious injury. There was severe morphological damage occurring in TBI rat brain tissues, with cellular rupture, light diffuse staining of cytoplasm, karyopyknosis, and hyper-staining. The brain tissues of the TBI + low, TBI + medium, and TBI + high groups revealed improvement in necrosis and lesions, whereas further aggravation of brain tissue injury in the TBI + Compound C group than those of TBI rats. We observed that there was ameliorated brain tissue injury occurred in rats of the TBI + high + Compound C group than those of the TBI + Compound C group. The scores of brain tissues in each group were recorded. The brain tissue score of TBI rats was higher than that of control rats (Figure 2a, *P* = 0.00, 95% CI −4.69, −1.97), whereas reduction occurred following UCF-101 addition (TBI vs TBI + low group: *P =* 0.23, 95% CI −0.36, 2.36; TBI vs TBI + medium group: *P* = 0.00, 95% CI 0.64, 3.36; TBI vs TBI + high group: *P* = 0.00, 95% CI 1.31, 4.03). The scores in the TBI + high + Compound C group exhibited a decrease compared to the TBI + Compound C group (Figure 2a, *P* = 0.012, 95% CI 0.31, 3.03).

The detection of BBB permeability was carried out by EB staining assay (Figure 2b). As displayed in Figure 2b, TBI rat brain tissues exhibited a clearly visible EB leakage and an increase in EB content than those of the control group (*P* = 0.00, 95% CI −24.12, −13.61). We intuitively observed that regardless of dosage of UCF-101 treatment, leakage of EB dye from the TBI rat brain tissues was attenuated, with lower levels of EB content (Figure 2b, TBI vs TBI + low group: *P* = 0.014, 95% CI 1.10, 11.61; TBI vs TBI + medium: *P* = 0.00, 95% CI 4.53, 15.03; TBI vs TBI + high group: *P* = 0.00, 95% CI 6.50, 17.01). In addition, EB content in the TBI + Compound C group was increased when compared to the TBI group (*P* = 0.049, 95% CI −10.53, −0.02), whereas lower EB content in the TBI + high + Compound C group than in the TBI + Compound C group (*P* = 0.00, 95% CI 2.51, 13.02). The assessment of brain edema was constructed by weighing and calculating the water content of rat brain tissues (Figure 2c). There was an enhanced cerebral edema index in TBI rat brain tissues than that of control rats (Figure 2c, *P* = 0.03, 95% CI 0.54, 12.76).

The measurement of BBB permeability-associated protein expression was made by Western blot assay (Figure 2d). The TBI group led to lower ZO-1, Occludin, and Claudin-5 expression levels in comparison to the control group (Figure 2d, *P* = 0.00, 95% CI −0.62, −0.39; 95% CI −0.66, −0.44; 95% CI −0.69, −0.42). Further UCF-101 treatment resulted in higher ZO-1, Occludin, and Claudin-5 expression in TBI rats (TBI vs TBI + low group: ZO-1: *P* = 0.00, 95% CI 0.17, 0.41; Occludin: *P* = 0.015, 95% CI 0.02, 0.24; Claudin-5: *P* = 0.00, 95% CI 0.07, 0.34; TBI vs TBI + medium group: ZO-1: *P* = 0.00, 95% CI 0.28, 0.52; Occludin: *P* = 0.00, 95% CI 0.09, 0.31; Claudin-5: *P* = 0.00, 95% CI 0.28, 0.55; TBI vs TBI + high group: ZO-1: *P* = 0.00, 95% CI 0.32, 0.55; Occludin: *P* = 0.00, 95% CI 0.15, 0.38; Claudin-5: *P* = 0.00, 95% CI 0.50, 0.77). The TBI + Compound C group exhibited lowered ZO-1, Occludin, and Claudin-5 protein expression in comparison to the TBI group (*P* = 0.00, 95% CI −0.34, −0.10; *P* = 0.00, 95% CI −0.30, −0.08; *P* = 0.025, 95% CI −0.29, −0.02). There was enhanced ZO-1, Occludin, and Claudin-5 protein expression in the TBI + high + Compound C group than in the TBI + Compound C group (*P* = 0.00, 95% CI 0.34, 0.57; 95% CI 0.14, 0.36; 95% CI 0.17, 0.44). Compound C intervention reversed the improvement of BBB permeability by UCF-101 in TBI rats.

### 4.3 UCF-101 treatment led to apoptosis inhibition in TBI rats

**Figure 3:**
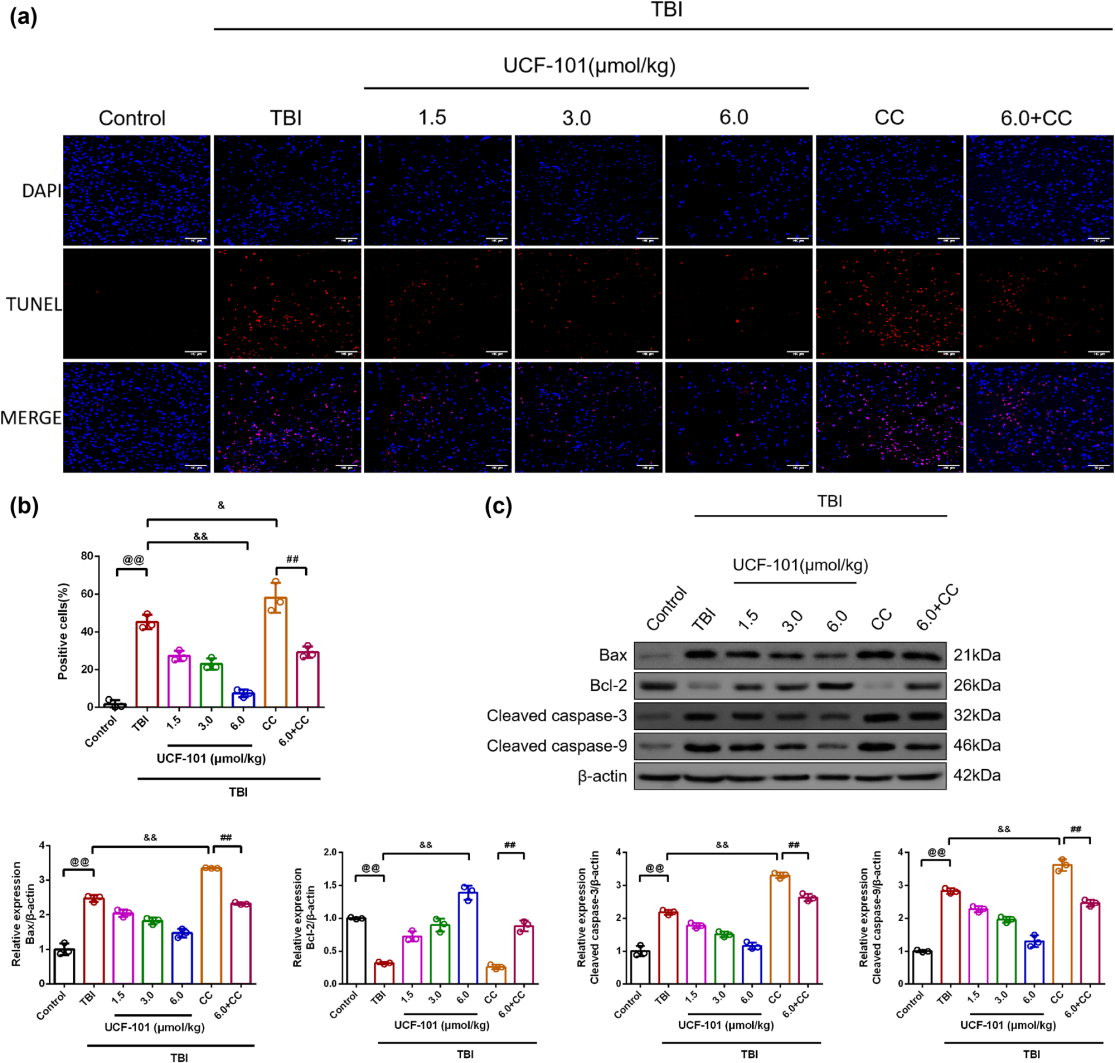
UCF-101 treatment led to apoptosis inhibition in TBI rats. (a and b) The apoptosis was measured by TUNEL staining (magnification 200×, scale bar: 100 μm), *n* = 3; (c) The protein expression of the Bax, Bcl-2, cleaved caspase-3, and cleaved caspase-9 in brain tissues was observed by Western blot, *n* = 3; ^@@^ *P* < 0.01 vs Control group, ^&^ *P* < 0.05 and ^&&^ *P* < 0.01 vs TBI group, ^##^ *P* < 0.01 vs TBI + Compound C group. Note: TBI: Traumatic brain injury; CC: Compound C; Bax: Bcl-2-associated X protein; cleaved caspase-3: cleaved cysteinyl aspartate specific proteinase-3; cleaved caspase-9: cleaved cysteinyl aspartate specific proteinase-9; TUNEL: Terminal deoxynucleotidyl transferase-mediated dUTP nick end labeling assay.

The determination of apoptosis was done by TUNEL staining assay, with apoptosis-related protein expression measured by Western blot (Figure 3). It was revealed that the apoptosis rate of TUNEL-positive neurons around the trauma site in TBI rats was higher compared to the control group (Figure 3a and b, *P* = 0.00, 95% CI −54.67, −32.40). The TBI + low, TBI + medium, and TBI + high groups displayed decreased TUNEL-positive neurons (*P* = 0.00, TBI vs TBI + low group: 95% CI 6.83, 29.10; TBI + medium group: 95% CI 11.09, 33.36; TBI vs TBI + high group: 95% CI 26.67, 48.94), whereas higher TUNEL-positive neurons caused by Compound C treatment (*P* = 0.02, 95% CI −23.96, −1.69). The apoptosis rate of the TBI + high + Compound C group was exhibited lower than that of rats in the TBI + Compound C group (*P* = 0.00, 95% CI 17.68, 39.96).

In Figure 3c, there were enhanced Bax, cleaved caspase-3, and cleaved caspase-9 expression, and a reduction in Bcl-2 protein expression in TBI group than in control rats (*P* = 0.00, 95% CI 1.18, 1.76; 95% CI 0.89, 1.48; 95% CI 1.50, 2.16; 95% CI −0.88, −0.48). Following low, medium, and high doses of UCF-101 intervention, there was lowered Bax, cleaved caspase-3, and cleaved caspase-9 expression, whereas higher Bcl-2 protein expression than those of TBI rats (TBI vs TBI + low group: Bax: *P* = 0.00, 95% CI −0.72, −0.14; cleaved caspase-3: *P* = 0.00, 95% CI −0.70, −0.11; cleaved caspase-9: *P* = 0.00, 95% CI −0.88, −0.22; Bcl-2: *P* = 0.00, 95% CI 0.20, 0.60; TBI vs TBI + medium group: Bax: *P* = 0.00, 95% CI −0.94, −0.36; cleaved caspase-3: *P* = 0.00, 95% CI −0.97, −0.38; cleaved caspase-9: *P* = 0.00, 95% CI −1.20, −0.54; Bcl-2: *P* = 0.00, 95% CI 0.37, 0.78; TBI vs TBI + high group: Bax: *P* = 0.00, 95% CI −1.29, −0.71; cleaved caspase-3: *P* = 0.00, 95% CI −1.32, −0.73; cleaved caspase-9: *P* = 0.00, 95% CI −1.86, −1.20; Bcl-2: *P* = 0.00, 95% CI 0.87, 1.27). Furthermore, we found that the TBI + Compound C group caused higher Bax, cleaved caspase-3, and cleaved caspase-9 expression when compared to the TBI group (*P* = 0.00, 95% CI 0.59, 1.17; 95% CI 0.83, 1.41; 95% CI 0.46, 1.12). There was reduced Bax, cleaved caspase-3, and cleaved caspase-9 protein expression, whereas increased Bcl-2 expression in the TBI + high + Compound C group than in the TBI + Compound C group (*P* = 0.00, 95% CI −1.32, −0.74; 95% CI −0.97, −0.38; 95% CI −1.48, −0.82; 95% CI 0.42, 0.82). Compound C treatment suppressed the inhibitory effects of UCF-101 on neuronal apoptosis in TBI rats.

### 4.4 UCF-101 treatment inhibited inflammation response through the AMPK/NF-κB pathway

**Figure 4:**
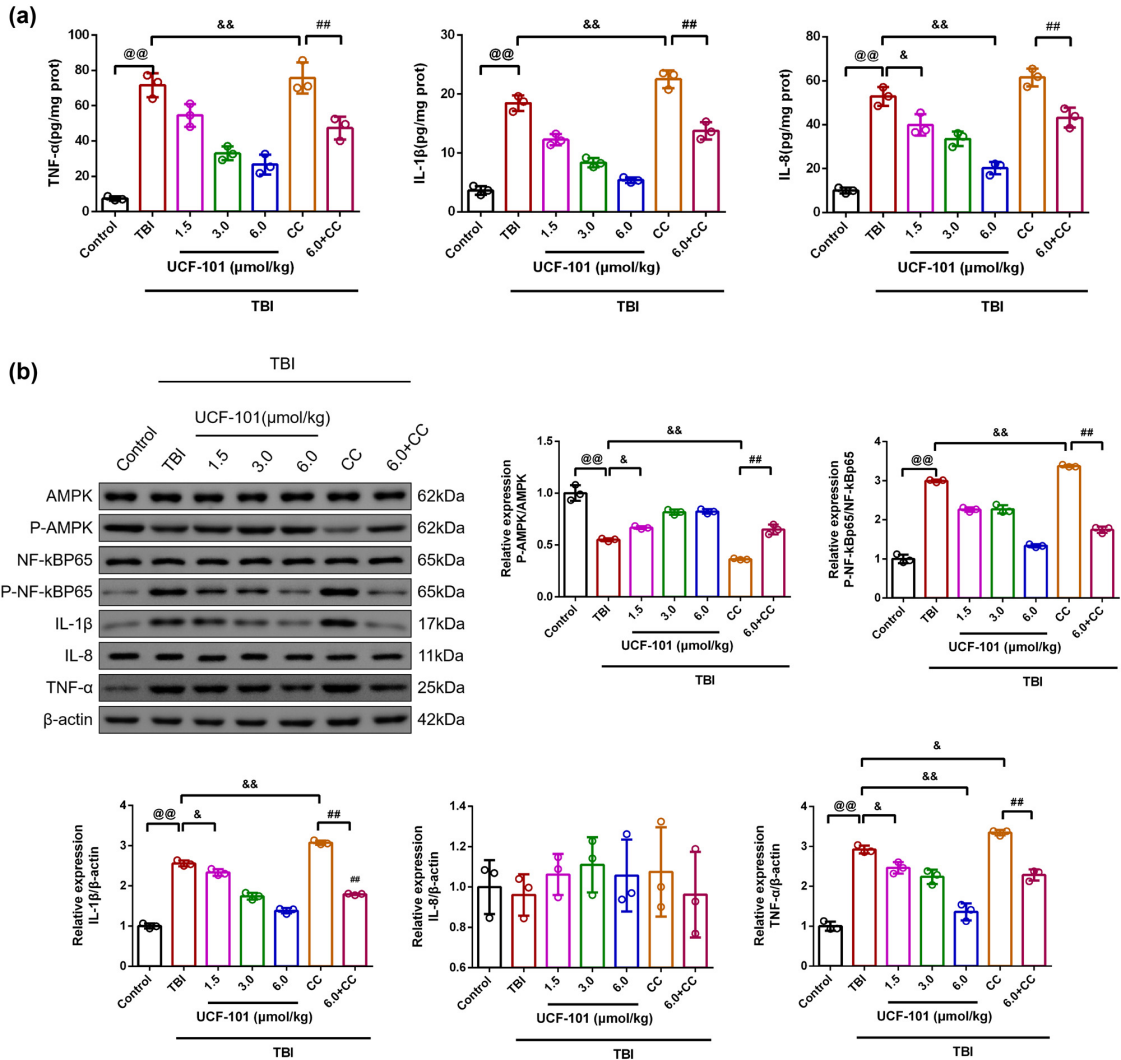
UCF-101 treatment inhibited inflammation response through the AMPK/NF-κB pathway. (a) The level of the TNF-α, IL-1β, and IL-8 in brain tissues of rats was determined using ELISA kits, *n* = 3. (b) The protein expression of the P-AMPK/AMPK, P-NF-κBp65/P-NF-κBp65, IL-1β, IL-8, and TNF-α in brain tissues was observed by Western blot, *n* = 3; ^@@^ *P* < 0.01 vs Control group, ^&^ *P* < 0.05 and ^&&^ *P* < 0.01 vs TBI group, and ^##^ *P* < 0.01 vs TBI + Compound C group. Note: CC: Compound C; TNF-α: tumor necrosis factor-α; IL-1β: interleukin-1β; IL-8: interleukin-8; AMPK/NF-κB: adenosine mono-phosphate activated protein kinase/nuclear factor-κB; ELISA: Enzyme-linked immunosorbent assay.

The inflammatory factors, including TNF-α, IL-1β, and IL-8 levels, were determined by ELISA (Figure 4a). There were higher TNF-α, IL-1β, and IL-8 levels in TBI rats than in control rats (*P* = 0.00, 95% CI 47.29, 80.95; 95% CI 11.73, 17.91; 95% CI 32.41, 53.30). Further UCF-101 intervention led to lowered TNF-α, IL-1β, and IL-8 expression in brain tissues of TBI rats (TBI vs TBI + low group: TNF-α: *P* = 0.047, 95% CI 0.16, 33.82; IL-1β: *P* = 0.00, 95% CI 3.12, 9.30; IL-8: *P* = 0.011, 95% CI 2.53, 23.43; TBI vs TBI + medium group: TNF-α: *P* = 0.00, 95% CI 21.74, 55.40; IL-1β: *P* = 0.00, 95% CI 7.02, 13.19; IL-8: *P* = 0.00, 95% CI 8.88, 29.77; TBI vs TBI + high group: TNF-α: *P* = 0.00, 95% CI 28.12, 61.78; IL-1β: *P* = 0.00, 95% CI 9.97, 16.15; IL-8: *P* = 0.00, 95% CI 22.20, 43.09). Following Compound C intervention in TBI rats, the IL-1β level exhibited increased (*P* = 0.00, 95% CI −20.89, 12.77; 95% CI −7.15, −0.98). In the TBI + high + Compound C group, TNF-α, IL-1β, and IL-8 levels in brain tissues displayed lowered in comparison to the TBI + Compound C group (*P* = 0.00, 95% CI 11.40, 45.06; 95% CI 5.67, 11.85; 95% CI 7.87, 28.77).

The detection of P-AMPK/AMPK, P-NF-κBp65/NF-κBp65, IL-1β, IL-8, and TNF-α protein expression was carried out by the Western blot method (Figure 4b). There were decreased P-AMPK/AMPK protein expression and enhanced P-NF-κBp65/NF-κBp65, IL-1β, TNF-α protein expression in TBI rats in comparison to control rats (*P* = 0.00, 95% CI −0.55, −0.35; 95% CI 1.79, 2.19; 95% CI 1.37, 1.74; 95% CI 1.52, 2.32). The TBI + low, TBI + medium, and TBI + high groups led to higher P-AMPK/AMPK expression and lower P-NF-κBp65/NF-κBp65, IL-1β, and TNF-α expression than those of the TBI group (TBI vs TBI + low group: TNF-α: *P* = 0.02, 95% CI −0.86, −0.06; IL-1β: *P* = 0.013, 95% CI −0.41, −0.04; P-AMPK/AMPK: *P* = 0.021, 95% CI 0.01, 0.22; P-NF-κBp65/NF-κBp65: *P* = 0.00, 95% CI −0.94, −0.54; TBI vs TBI + medium group: TNF-α: *P* = 0.00, 95% CI −1.09, −0.29; IL-1β: *P* = 0.00, 95% CI −1.00, −0.63; P-AMPK/AMPK: *P* = 0.00, 95% CI 0.16, 0.37; P-NF-κBp65/NF-κBp65: *P* = 0.00, 95% CI −0.93, −0.53; TBI vs TBI + high group: TNF-α: *P* = 0.00, 95% CI −1.96, −1.16; IL-1β: *P* = 0.00, 95% CI −1.36, −0.10; P-AMPK/AMPK: *P* = 0.00, 95% CI 0.17, 0.38; P-NF-κBp65/NF-κBp65: *P* = 0.00, 95% CI −1.86, −1.46). Meanwhile, the TBI + Compound C group exhibited higher expression of P-NF-κBP65, IL-1β, and TNF-α, whereas lower P-AMPK/AMPK expression than in the TBI group (*P* = 0.00, 95% CI 0.18, 0.58; *P* = 0.00, 95% CI 0.33, 0.70; *P* = 0.00, 95% CI; *P* = 0.00, 95% CI −0.29, −0.08; *P* = 0.035, 95% CI 0.02, 0.82). Additionally, we found that there was higher expression of P-AMPK/AMPK, and lowered protein expression of P-NF-κBp65/NF-κBp65, IL-1β, and TNF-α in the TBI + high + Compound C group than those of the TBI + Compound C group (*P* = 0.00, 95% CI 0.18, 0.39; 95% CI −1.83, −1.43; 95% CI −1.47, −1.10; 95% CI −1.46, −0.66).

## 5 Discussion

Injuries occurring in TBI can be categorized as primary mechanical injuries and delayed secondary injuries [27]. Secondary injuries lead to neuronal dysfunction, metabolic changes, neuroinflammation, BBB disruption, and brain edema [28]. Therefore, delayed secondary injuries offer potential targets for therapeutic interventions, which have attracted widespread attention [27]. In this study, we found that UCF-101 intervention with low, medium, and high doses exhibited neuroprotective effects on TBI rats to different degrees. UCF-101 treatment performed inhibition of inflammatory response and apoptosis by regulating the AMPK/NF-κB pathway, thereby ameliorating neurological impairments, cognitive function, and BBB permeability, alleviating cerebral edema, and consequently leading to TBI improvement.

UCF-101 was intraperitoneally injected into rats at doses of 1.5 μmol/kg and 10 μmol/kg in previous studies [16,22]. The dose of UCF-101 used for intraperitoneal injection was 10 μmol/kg in septicemia encephalopathy rats and 1.5 μmol/kg in cerebral ischemia rats [22]. In our study, three doses of UCF-101, 1.5, 3.0, and 6.0 μmol/kg were intraperitoneally injected for TBI rats, with dose-dependent neuroprotective effects. However, the optimal dose of UCF-101 treatment has yet to be investigated.

The majority of individuals with TBI exhibit symptoms of cognitive degradation, attention deficit, depression, and fatigue [29]. In animal models, sequelae of TBI are primarily characterized as impaired learning and memory capabilities and psychiatric ailments [30,31]. Our findings demonstrated that TBI rats exhibited briefer stepping into the incubation period, a larger number of mistakes, and diminished cognitive function. UCF-101 therapy noticeably enhanced cognitive function. It was found that flurbiprofenate mitigated cognitive function in rats with mild cognitive impairment via the AMPK/NF-κB signaling pathway [32]. In our investigation, the addition of an AMPK inhibitor, Compound C, resulted in cognitive impairment in TBI rats, which was improved following high-dose UCF-101, further supporting the moderating influence of the AMPK/NF-κB signaling pathway in the neurological damage recovery of UCF-101 treatment for TBI rats.

The BBB disruption and concomitant inflammatory response are direct consequences of TBI triggering which can last for an extended period of time [33]. Tight junction proteins, including Occludin, Claudins, and ZO-1, are essential for BBB integrity [34]. Hydroxysafflor yellow A was found to improve TBI by inhibition of BBB permeability, inflammation, and apoptosis, with up-regulating Occludin, Claudin-1, and ZO-1 expression, and reducing IL-1β, IL-6, TNF-α, NF-κB, Bax, caspase-3, as well as caspase-9 expression [25]. The betulinic acid hydroxamate, VCE-005.1, decreased neuronal apoptosis and preserved BBB integrity through activation of AMPK expression, thereby improving TBI [35]. The results of the above studies remained highly consistent with our findings. In our study, the AMPK inhibitor, Compound C, reversed the above expression in TBI rats, and high-dose UCF-101 treatment eliminated this effect, indicating that the AMPK/NF-κB pathway was one of the key targets for mitigating TBI development with UCF-101 treatment. Of note, TBI-induced BBB injury further contributed to increased extravasation of immune cells, promoting the development of secondary injury [36]. The astrocytes, microglia, and monocytes have been proven to be associated with BBB dysfunction and impaired homeostasis in TBI [36,37]. Astrocyte activation promotes synaptic remodeling, tissue repair, and neuronal survival after TBI [37]. Jiang et al. [36] indicated that Phillyrin exerted anti-TBI activity by promoting M2 polarization and inhibiting M1 polarization in microglia to ameliorate BBB injuries. Recent research has identified that as molecules regulating astrocyte function, ETB receptor, H2 receptor, and TRPV4 may be potential candidates for TBI therapy, and exploration of therapeutic medications for TBI has focused on functional molecules in astrocytes [38]. Moreover, microglia-mediated neuroinflammatory response after TBI exacerbated neuronal injury [39]. Therefore, further exploration of specific mechanisms of astrocytes and microglia in TBI neuroinflammation contributes to the development of new TBI therapeutics.

The limitations of our study need to be discussed here. Our study only focused on one of the downstream targets of UCF-101, and the signaling pathways involved in TBI are intertwined and complex, and further in-depth studies are still needed regarding whether it can exert its neuroprotective effects from other signaling pathways. The CCI method has been widely applied to develop TBI models, it only creates focal injuries and is limited to produce diffuse damage despite its advantages in reproducibility and high precision. There are numerous methods for the construction of TBI animal models, and significant heterogeneity is one of the biggest challenges. A strategy to overcome this problem could be to attempt to apply complementary models, pooling the simulation strengths of each model. The construction of animal models deepens our understanding of TBI, systematically explores the pathogenic mechanisms of TBI, and performs tightly controlled experiments to evaluate new diagnostic and therapeutic approaches providing the necessary tools that will ultimately lead to more informed medical decisions and better treatment.

In conclusion, Omi/HtrA2 inhibitor, UCF-101, may inhibit apoptosis, inflammatory response, and BBB permeability to exert neuroprotective effects in TBI rats by regulating AMPK/NF-κB pathway, offering new promising targets for TBI treatment and advancing UCF-101 clinical applications for TBI treatment.

## Supplementary Material

Supplementary material-1

Supplementary material-2
