# Supplementary material for: High-temperature requirement serine protease A2 inhibitor UCF-101 ameliorates damaged neurons in traumatic brain-injured rats by the AMPK/NF-κB pathway
Source: Open Life Sci. 2025 Mar 6;20(1):20220971. doi: 10.1515/biol-2022-0971 (PMC11889502; doi:10.1515/biol-2022-0971)
Supplement: Supplementary material-1 [file biol-2022-0971-sm1.pdf]

1. ZO-1, Occludin, and Claudin-5 in rat brain tissues
2. P-NF-kBP65, IL-1β, and TNF-α in rat brain tissues
3. Bax, Cleaved caspase-3, Cleaved caspase-9, and Bcl-2 in rat brain tissues

1. ZO-1, Occludin, and Claudin-5 in rat brain tissues

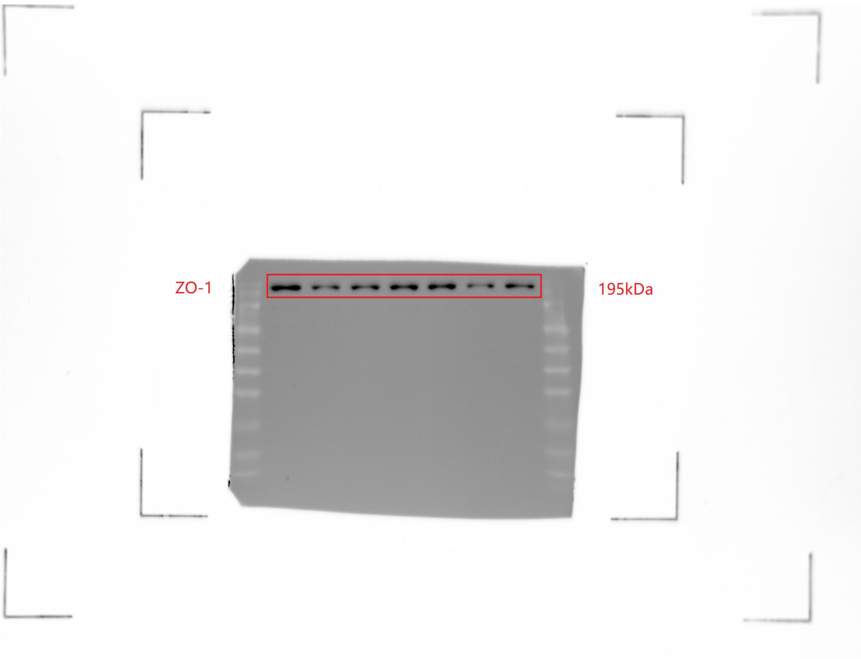

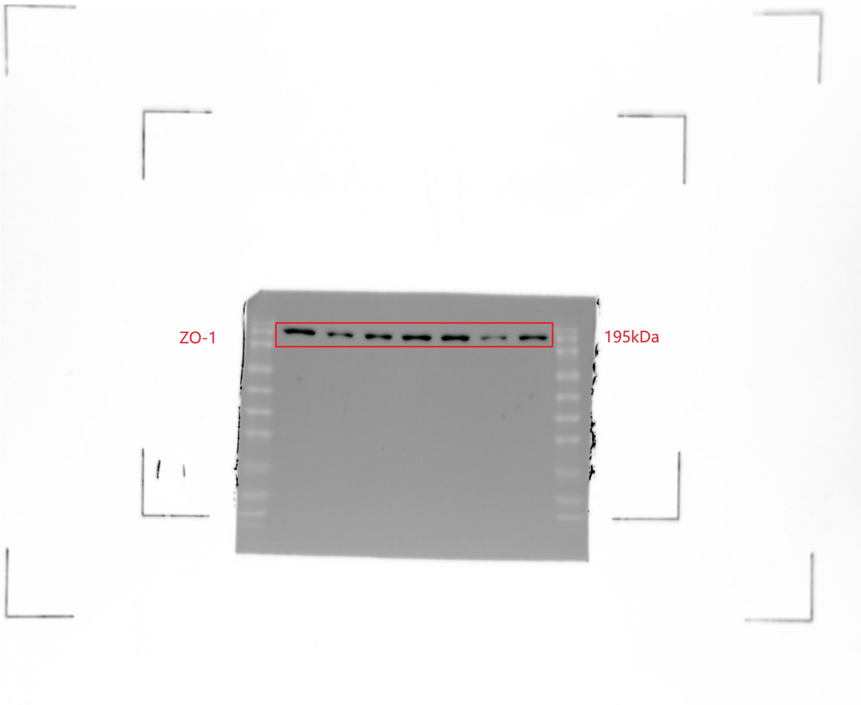

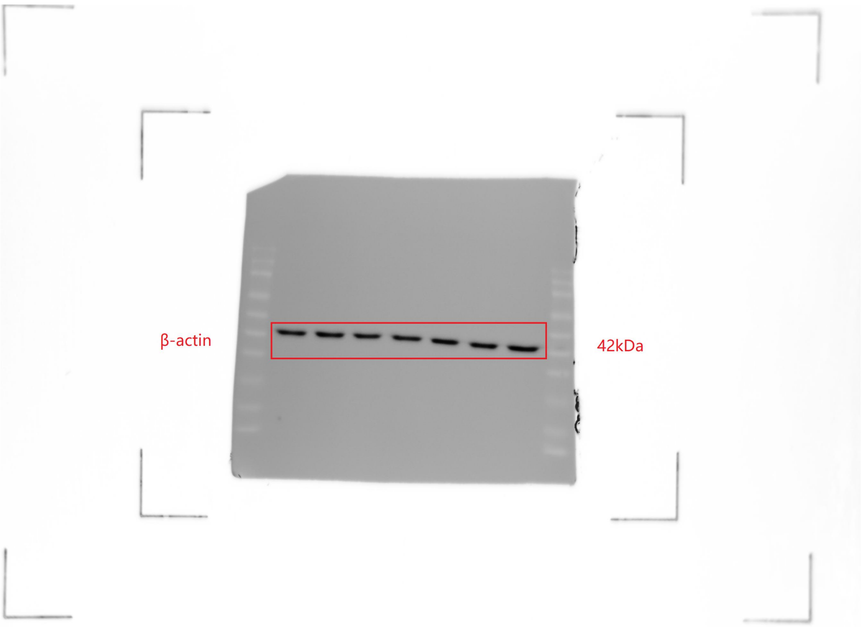
β-actin
42kDa

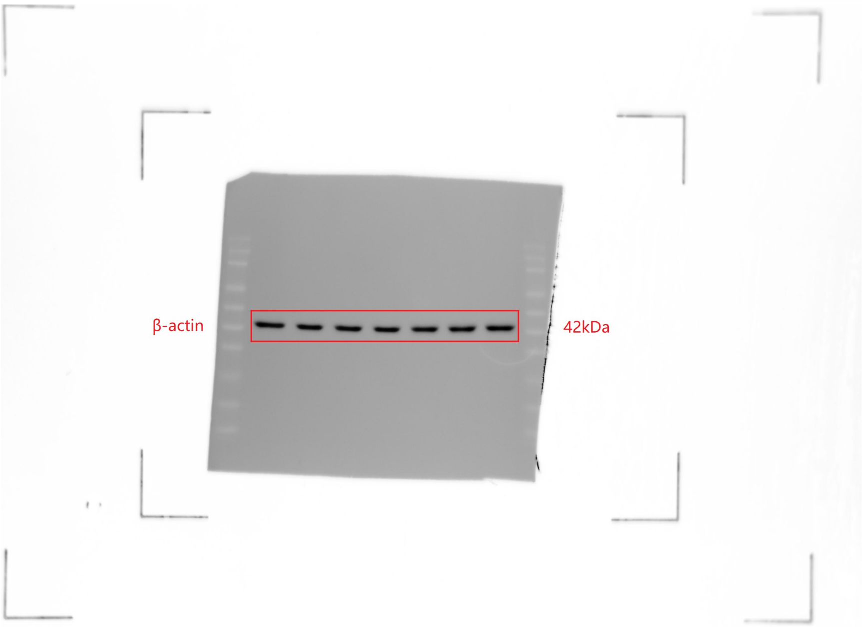
β-actin
42kDa

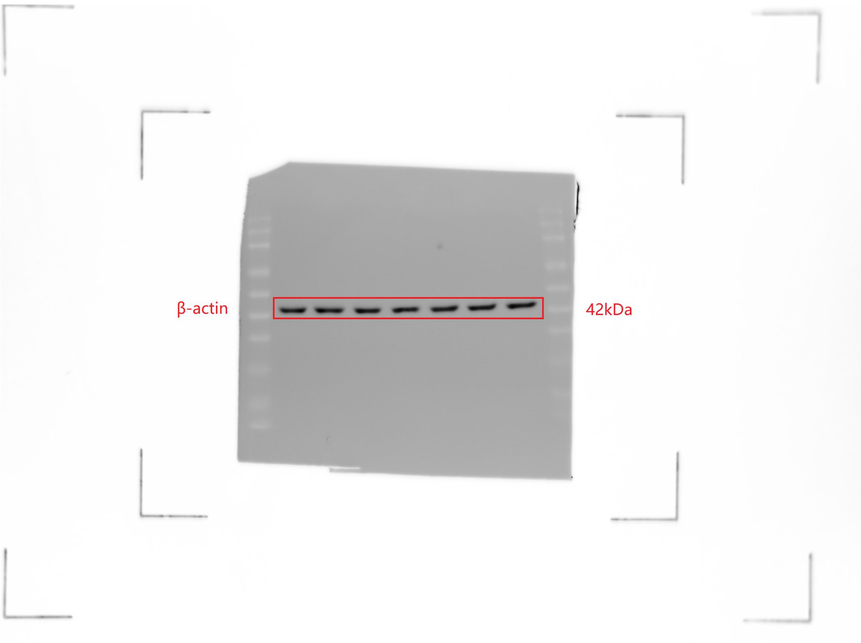
β-actin
42kDa

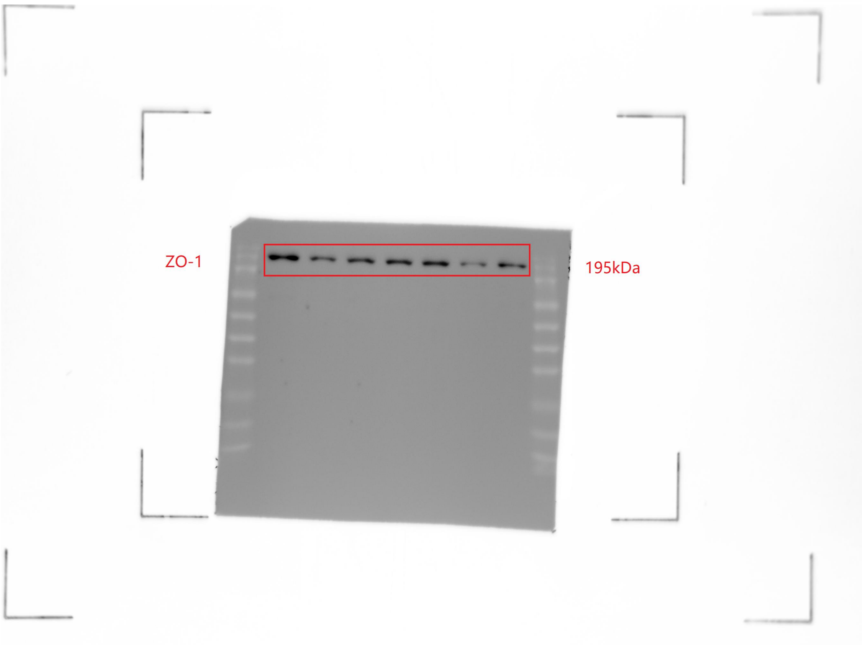
ZO-1
195kDa

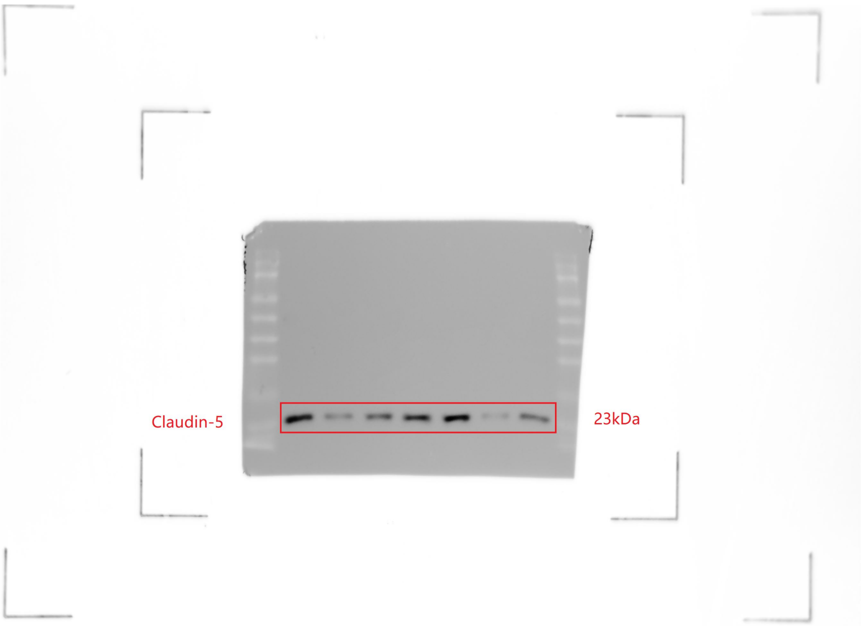
Claudin-5
23kDa

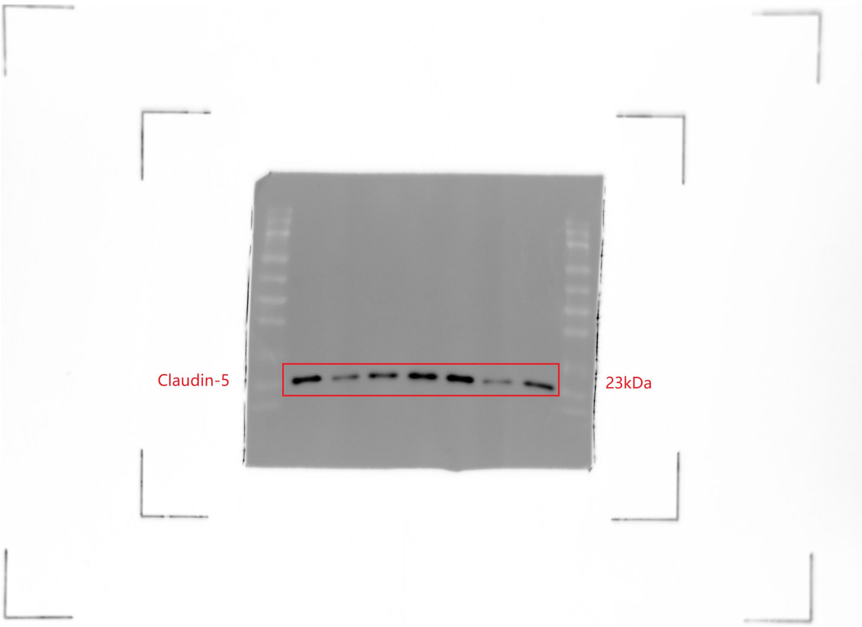
Claudin-5
23kDa

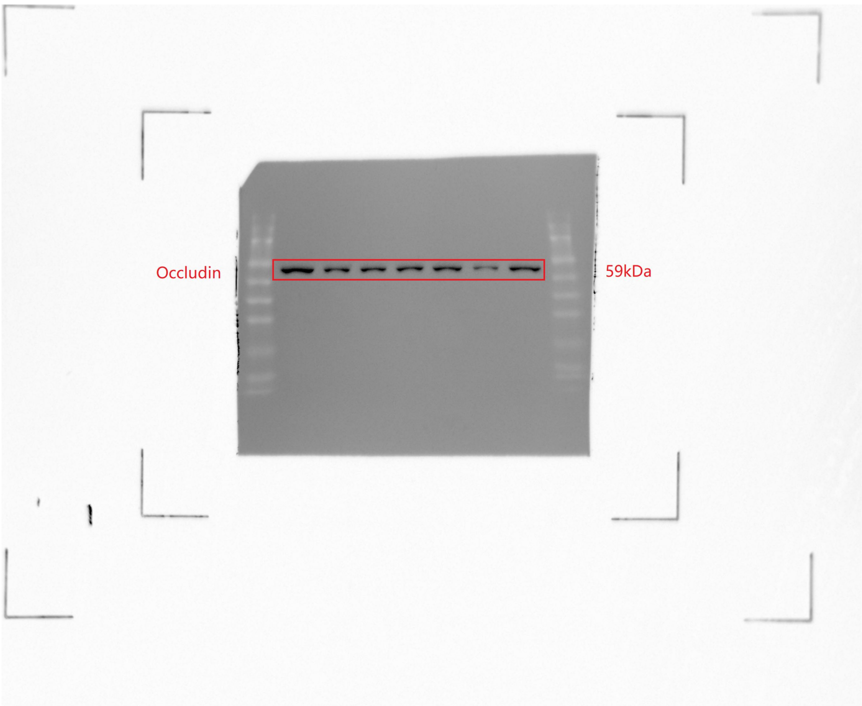
Occludin
59kDa

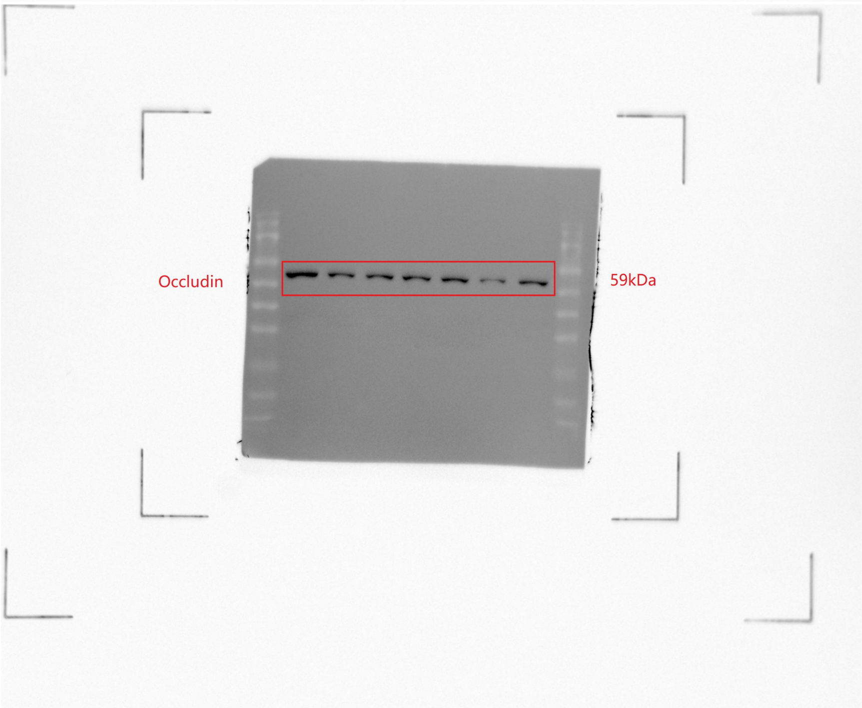
Occludin
59kDa

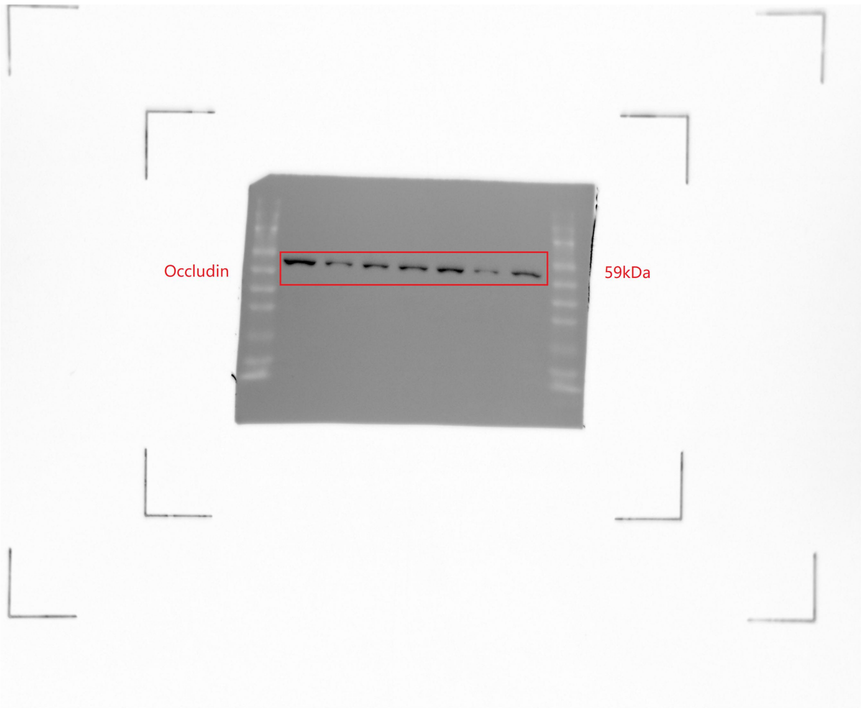

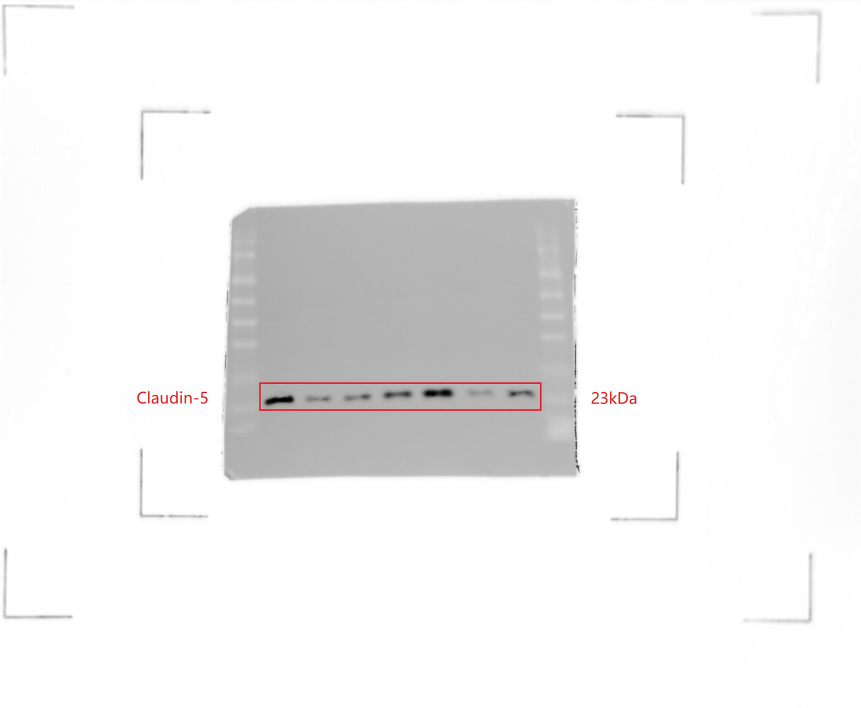

2. P-NF-kBP65, IL-1β, and TNF-α in rat brain tissues

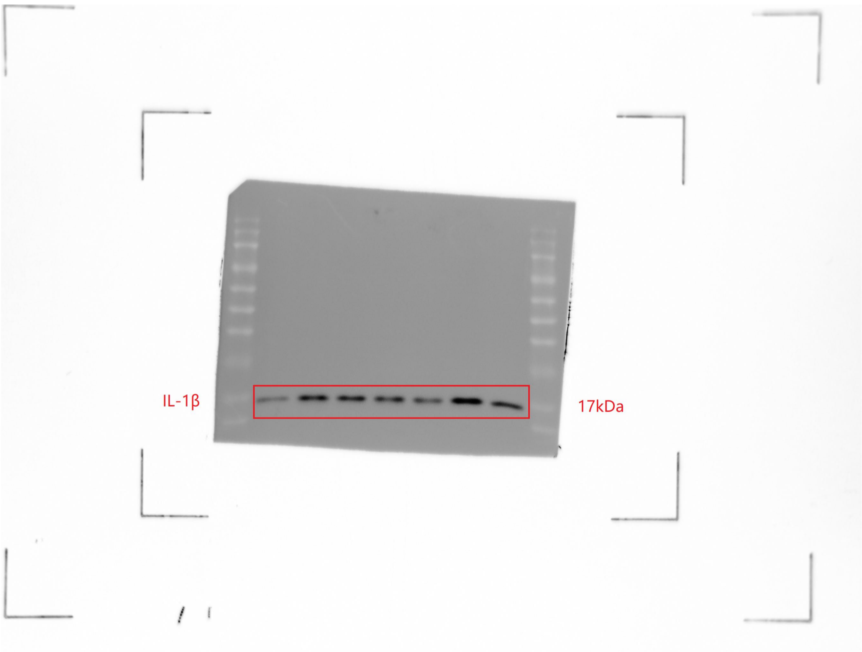
IL-1β
17kDa

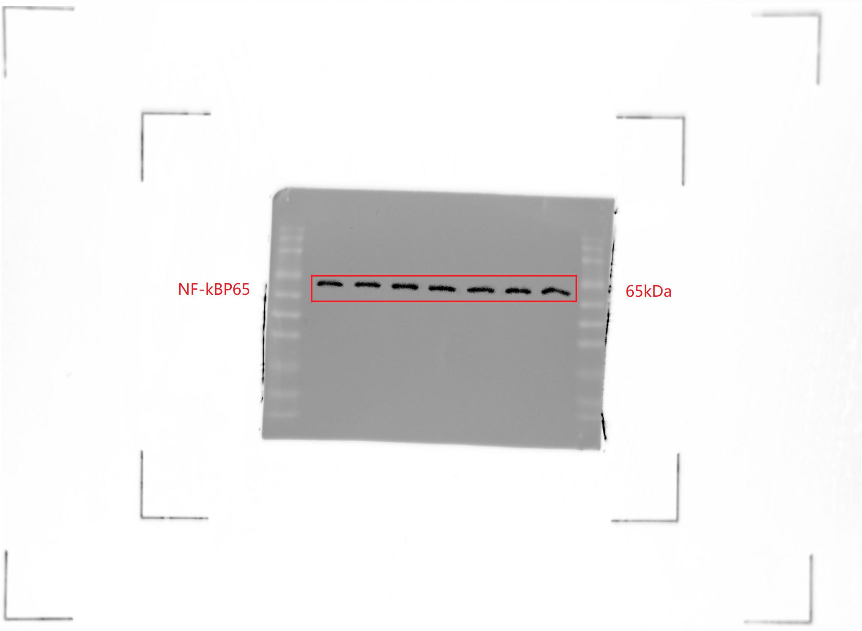
NF-kBP65
65kDa

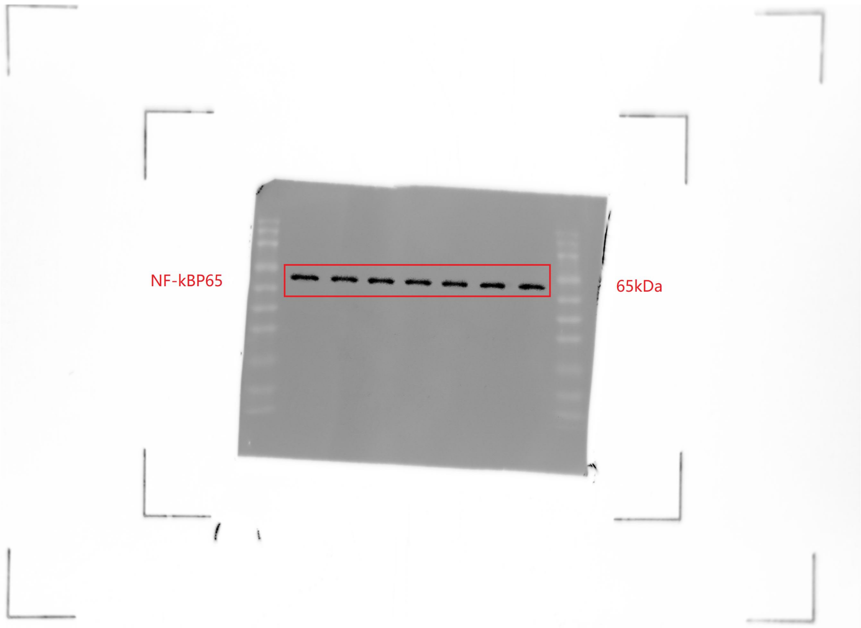
NF-kBP65
65kDa

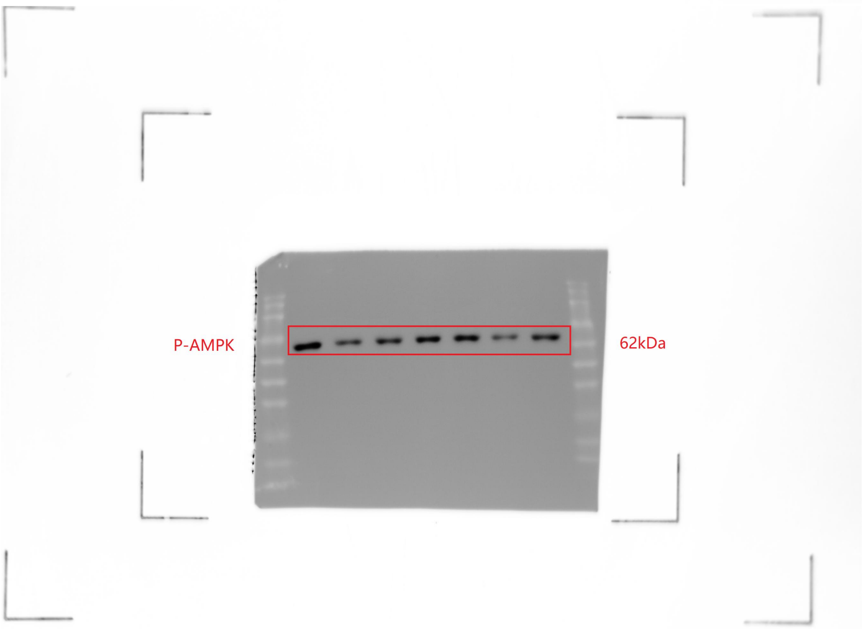
P-AMPK
62kDa

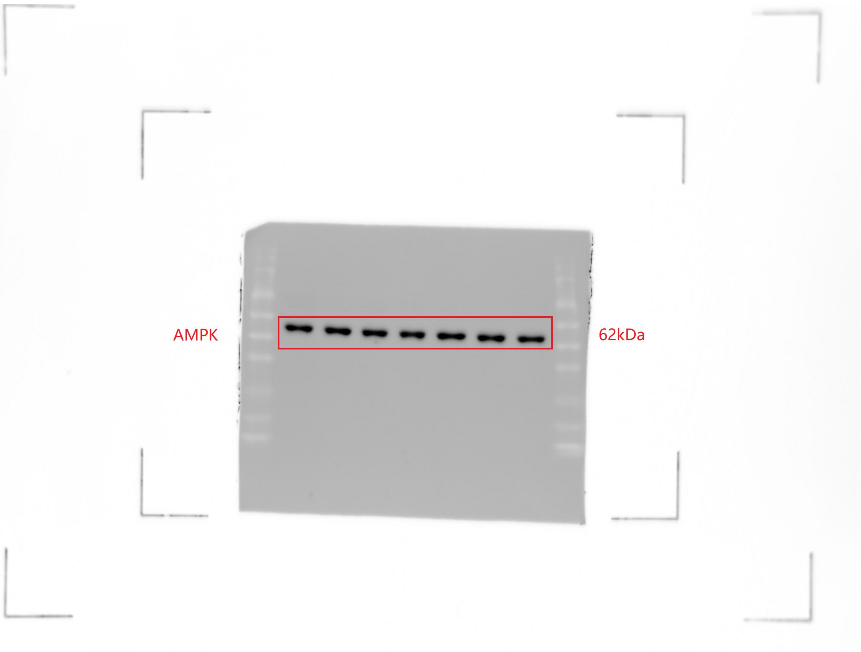
AMPK
62kDa

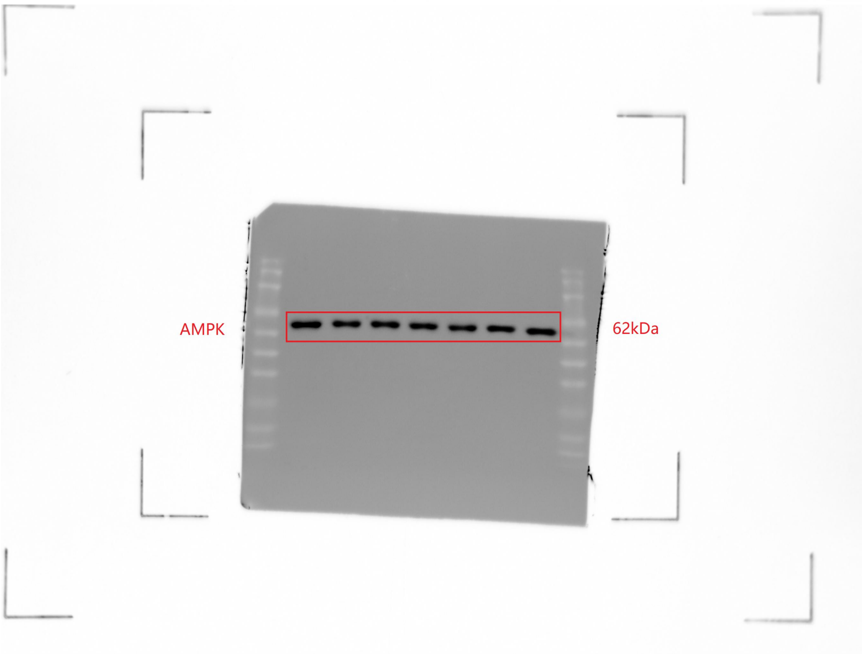
AMPK
62kDa

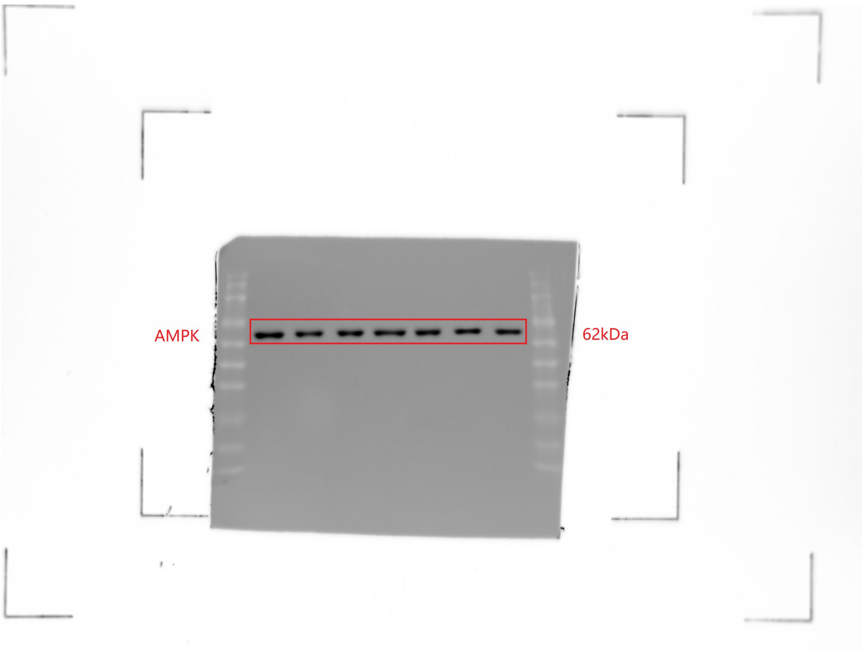
AMPK
62kDa

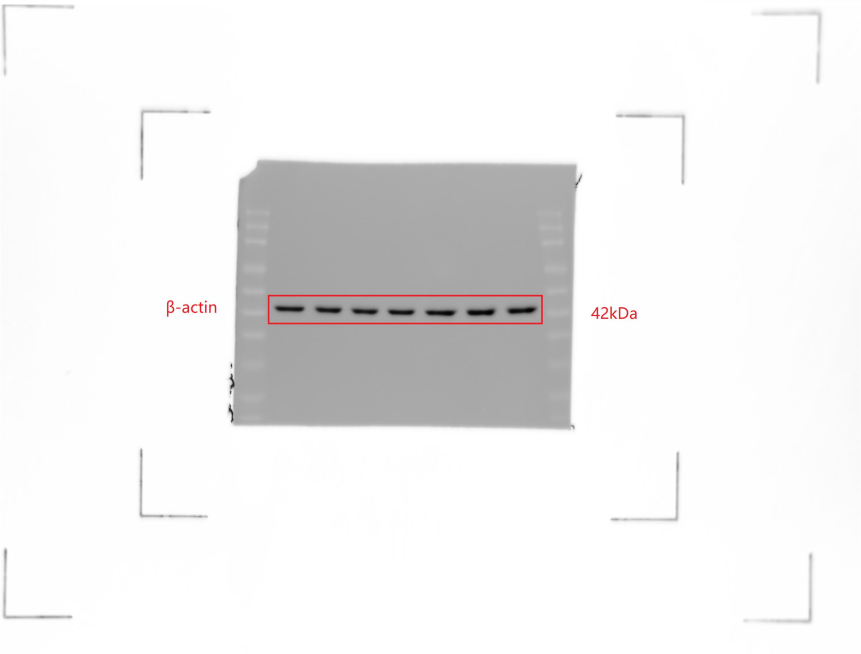
β-actin
42kDa

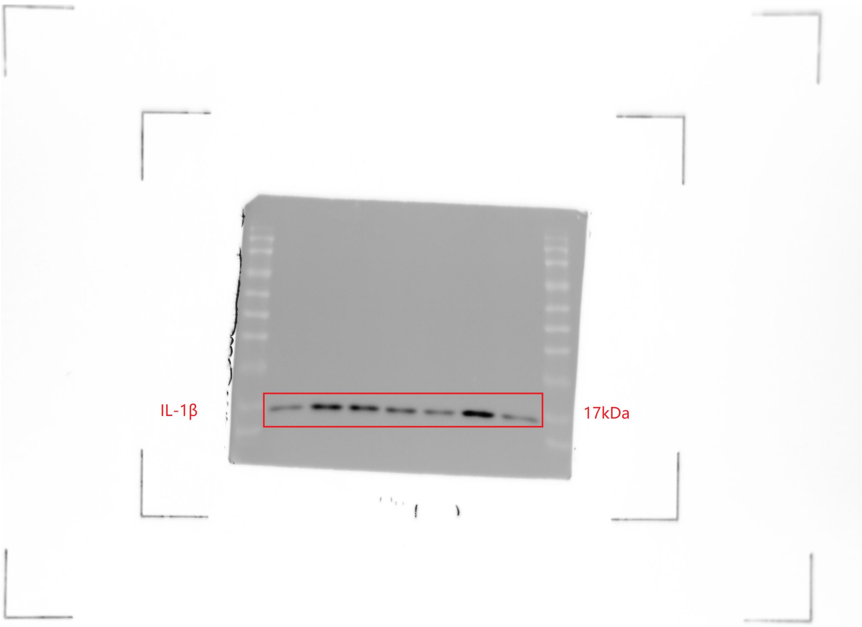
IL-1β
17kDa

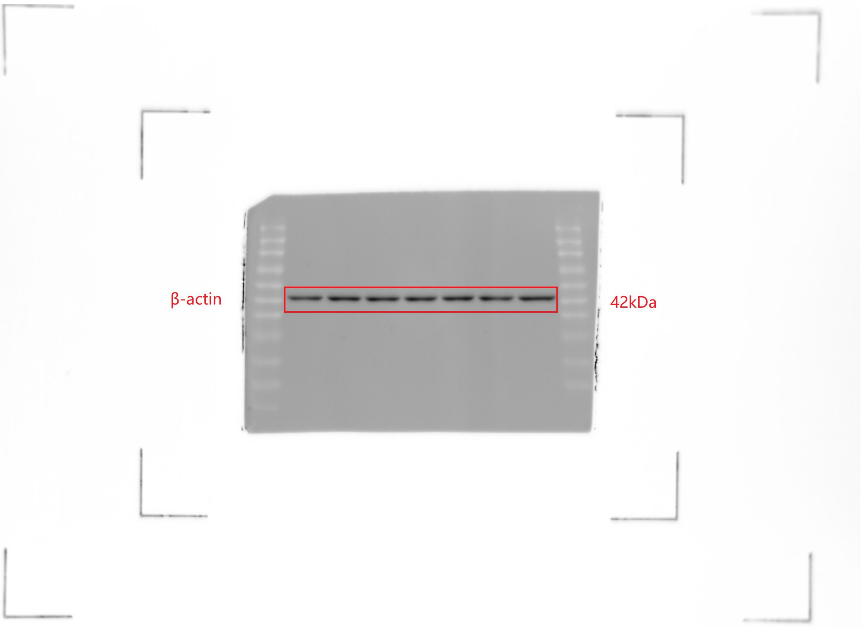
β-actin
42kDa

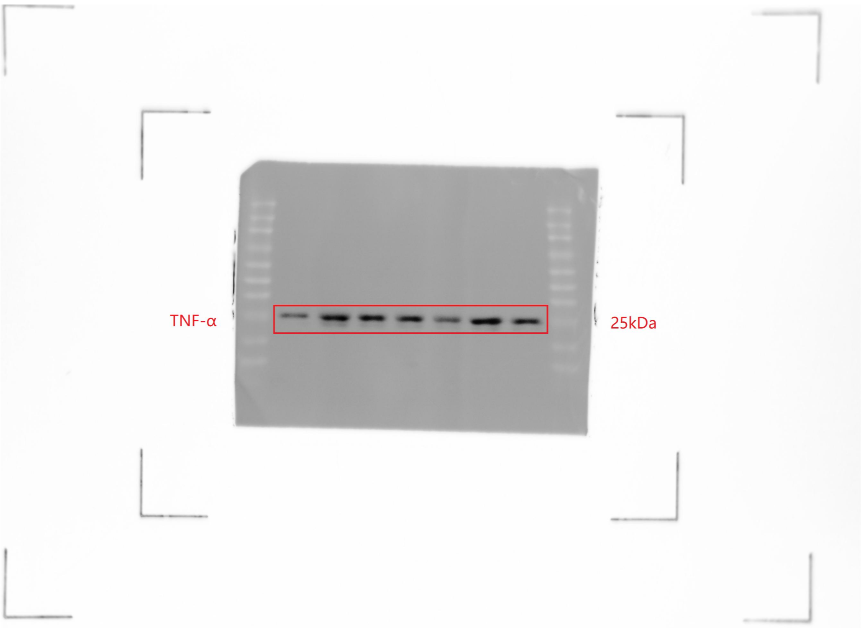
TNF-α
25kDa

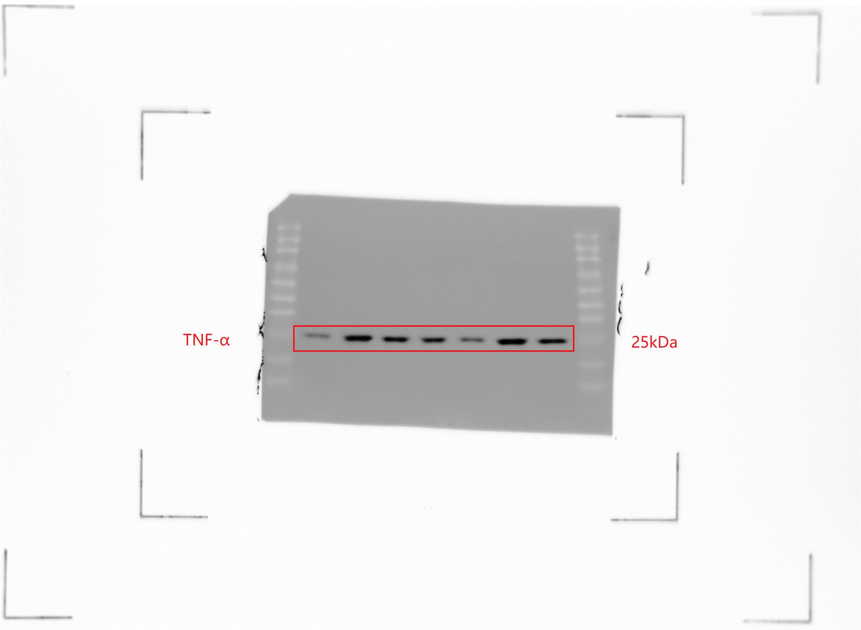
TNF-α
25kDa

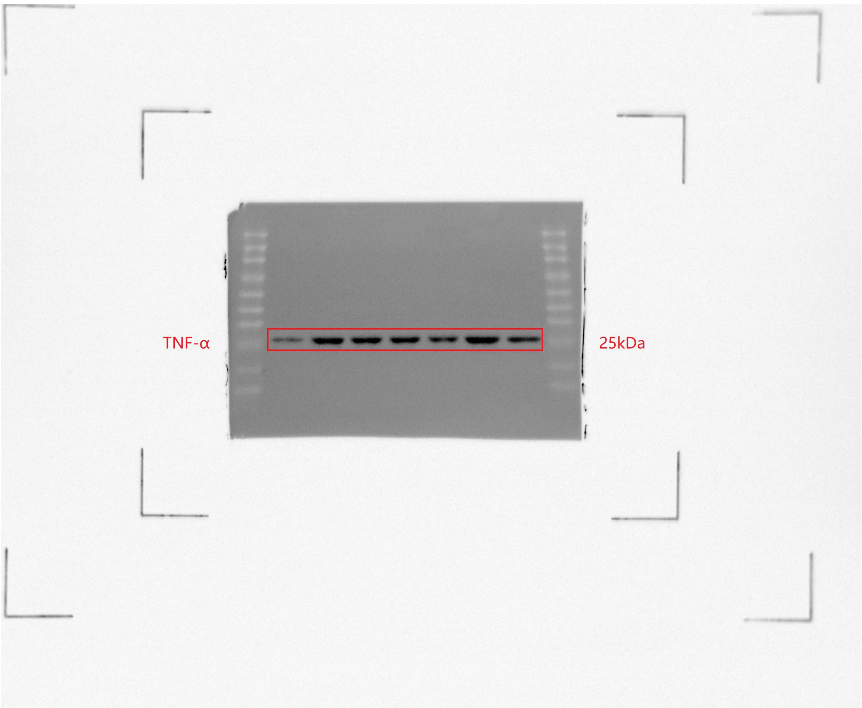
TNF-α
25kDa

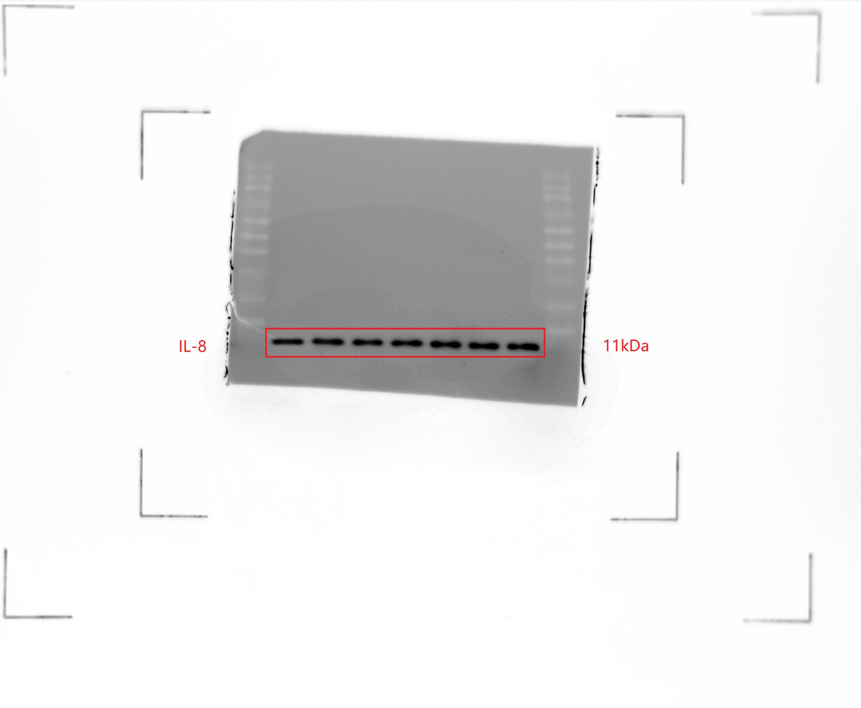
IL-8
11kDa

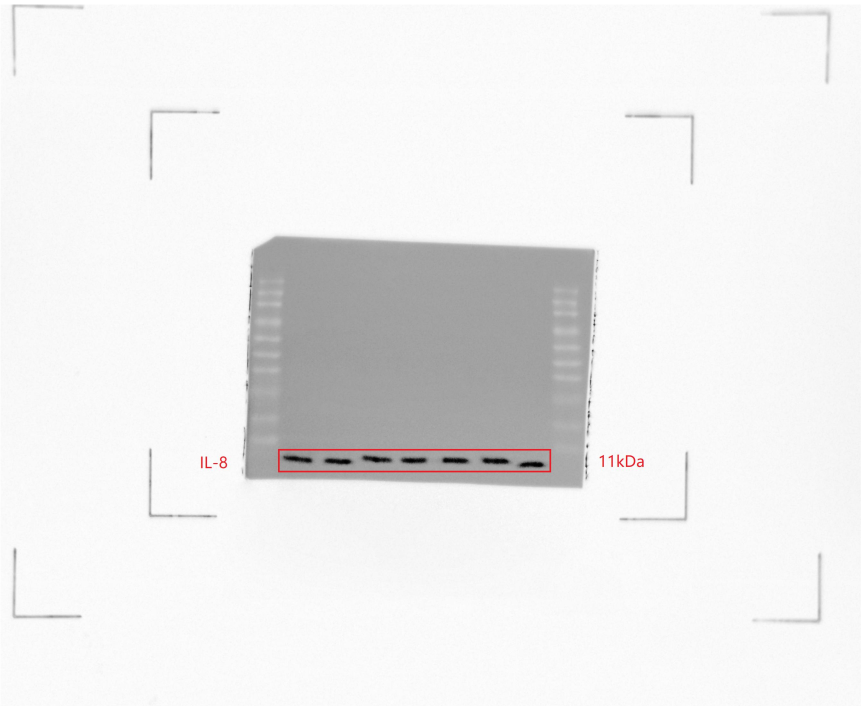
IL-8
11kDa

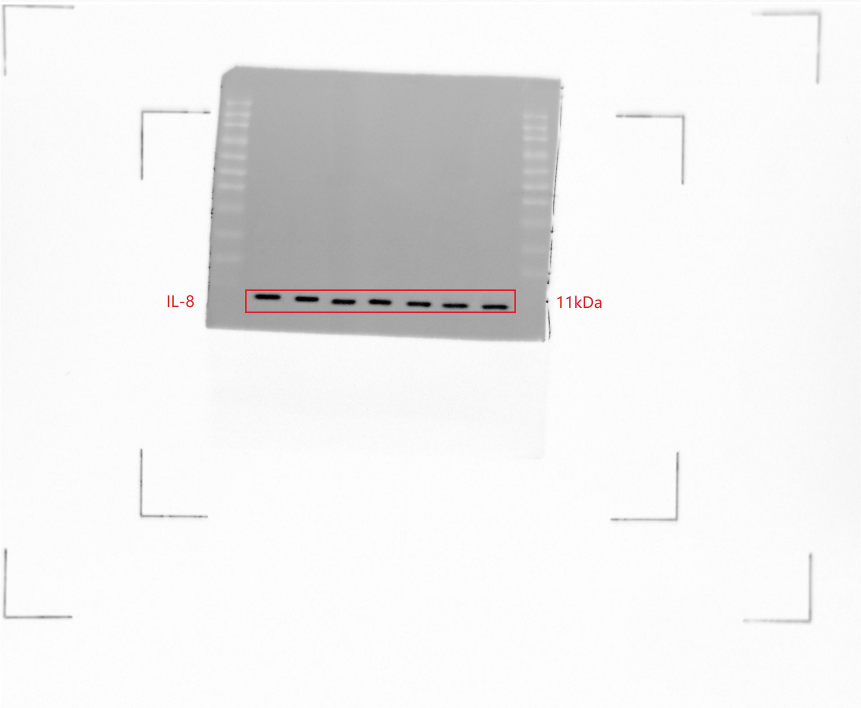
IL-8
11kDa

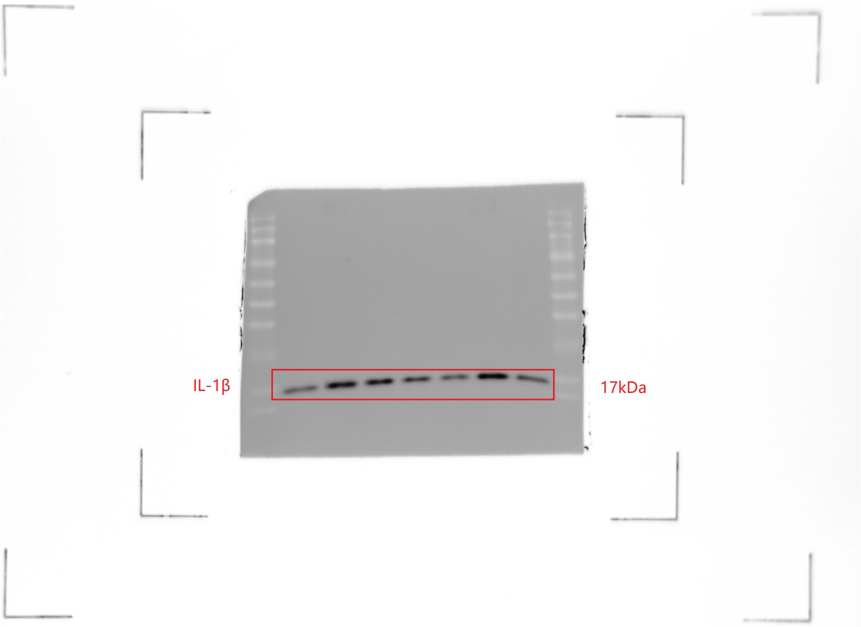
IL-1β
17kDa

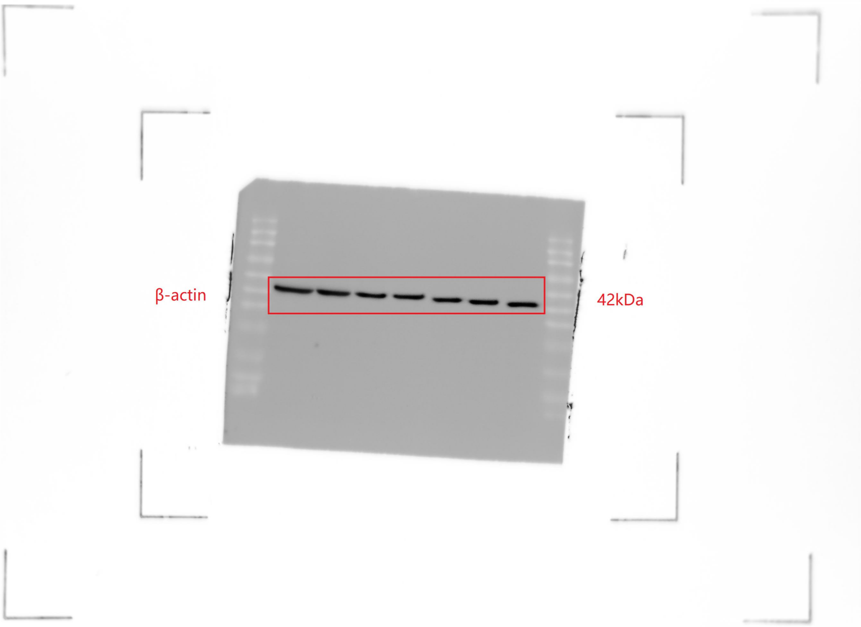
β-actin
42kDa

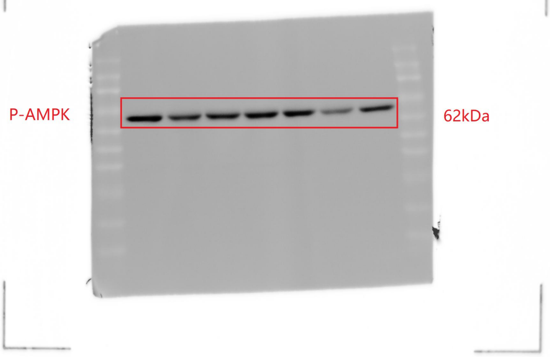
P-AMPK
62kDa

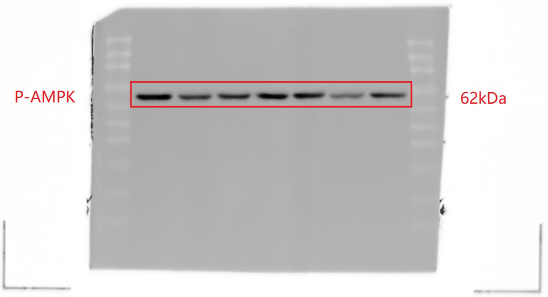
P-AMPK
62kDa

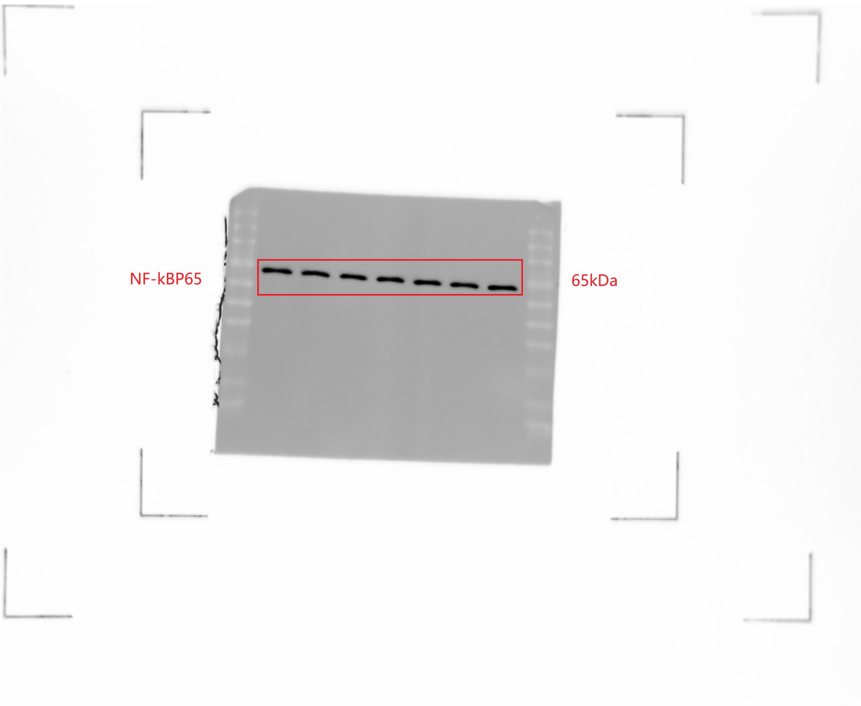
NF-kBP65
65kDa

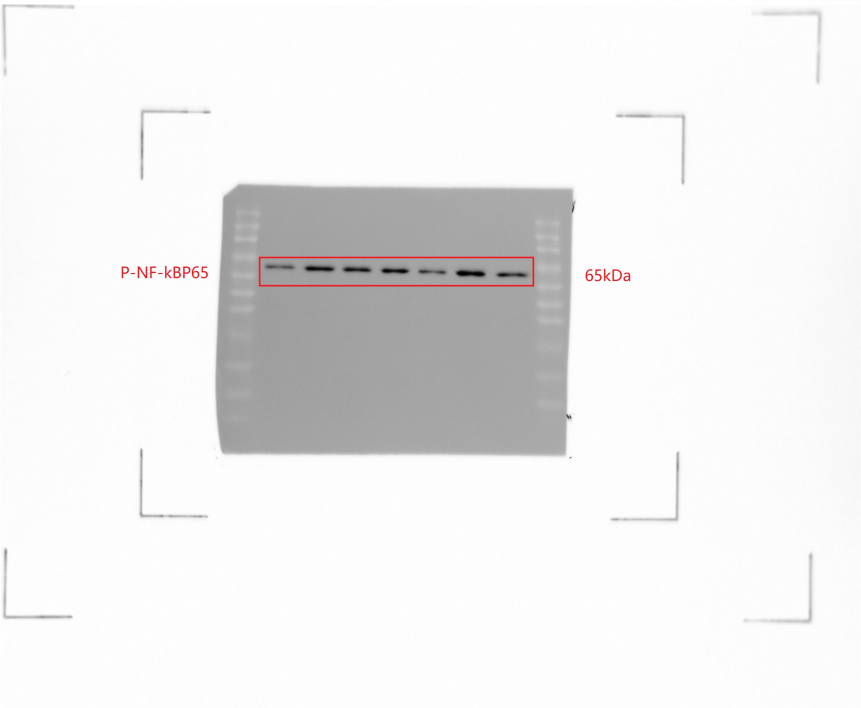
P-NF-kBP65
65kDa

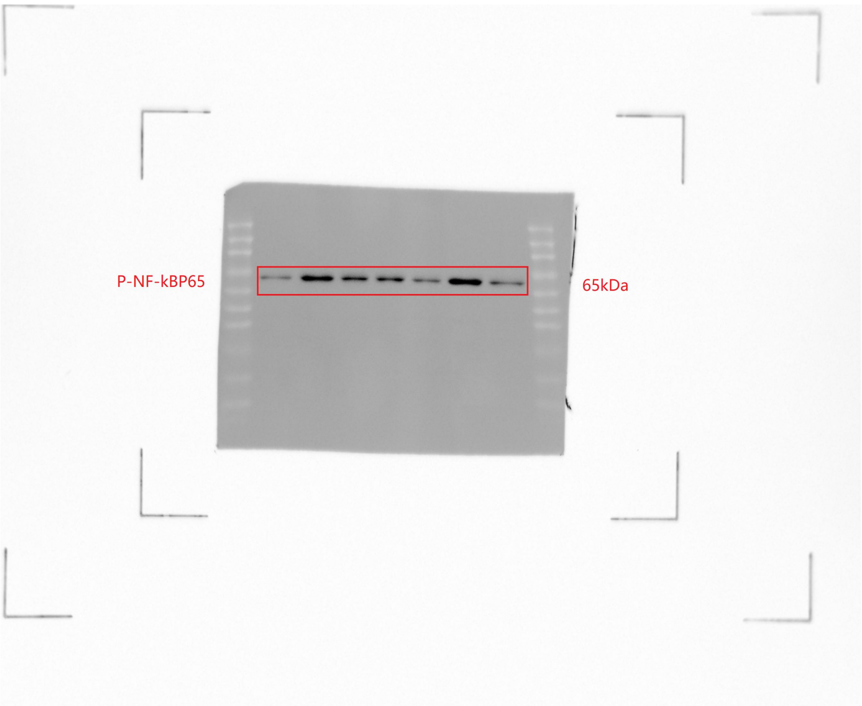

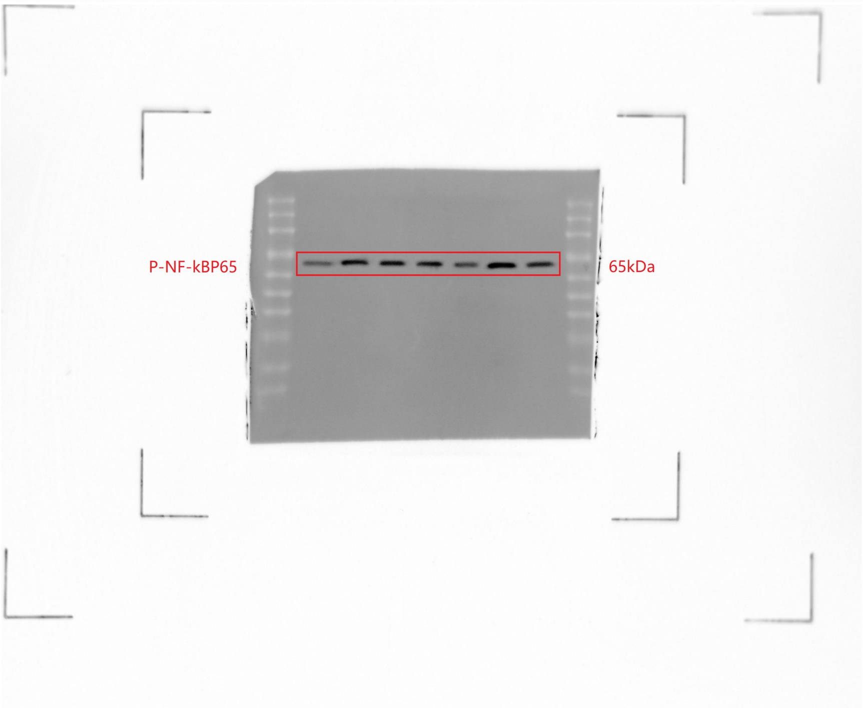

3. Bax, Cleaved caspase-3, Cleaved caspase-9, and Bcl-2 in rat brain tissues

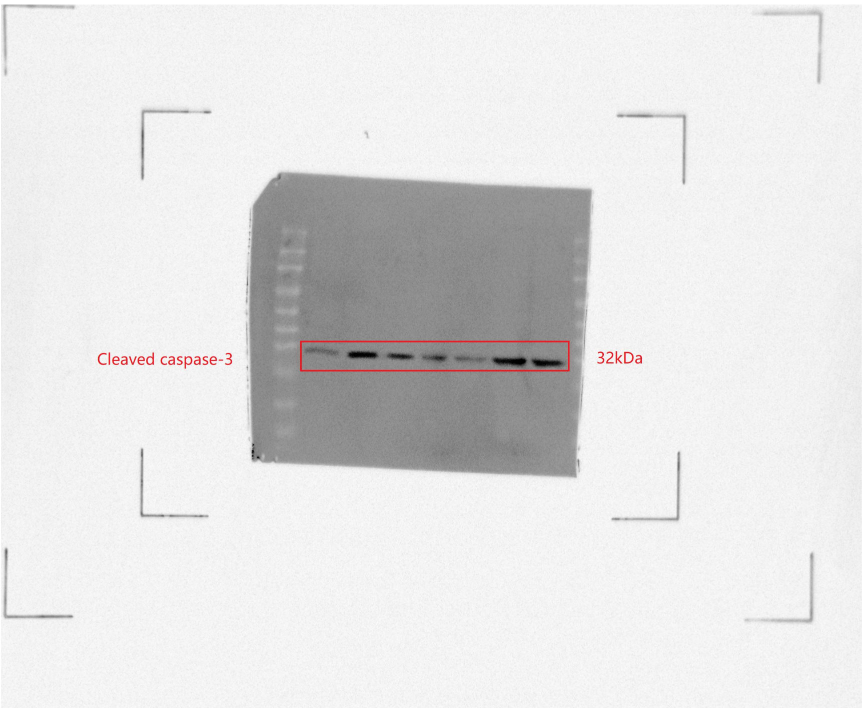
Cleaved caspase-3
32kDa

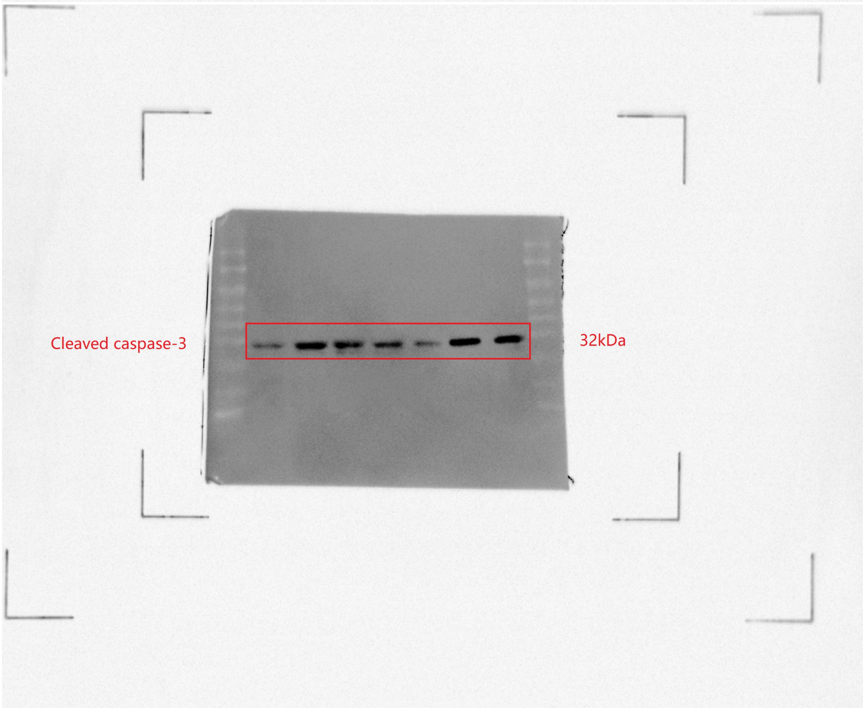
Cleaved caspase-3
32kDa

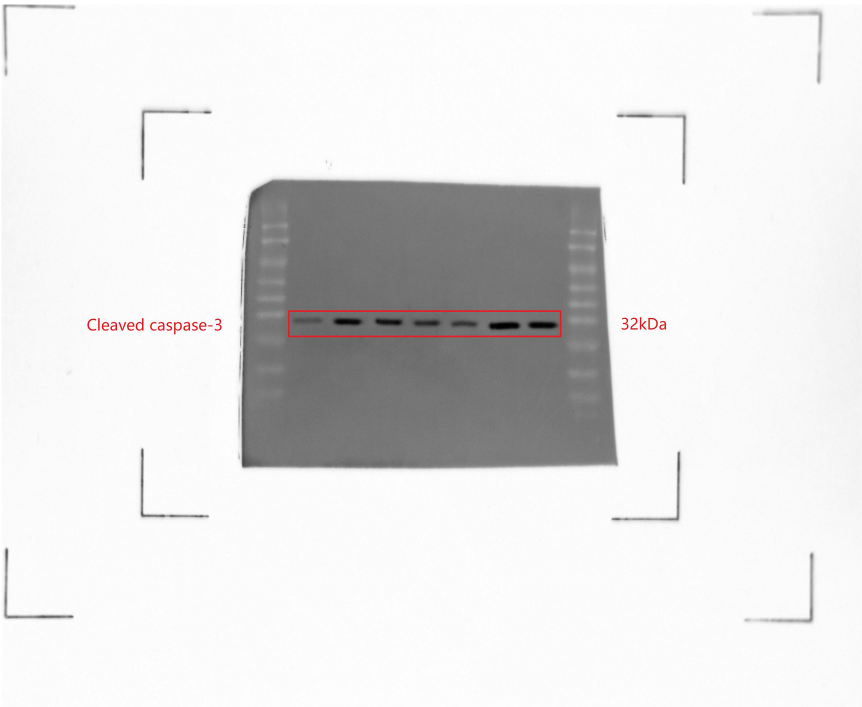
Cleaved caspase-3
32kDa

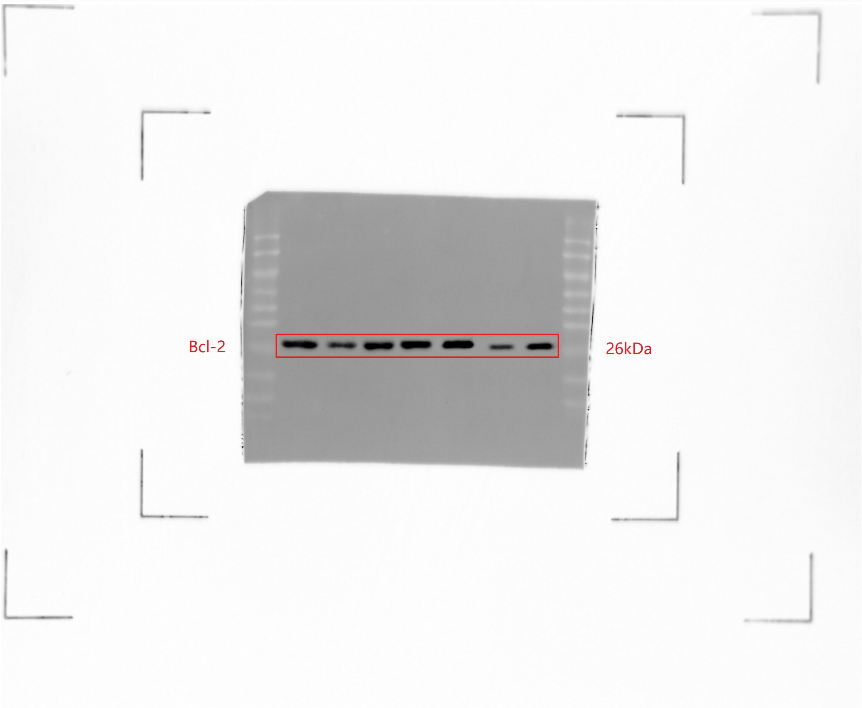
Bcl-2
26kDa

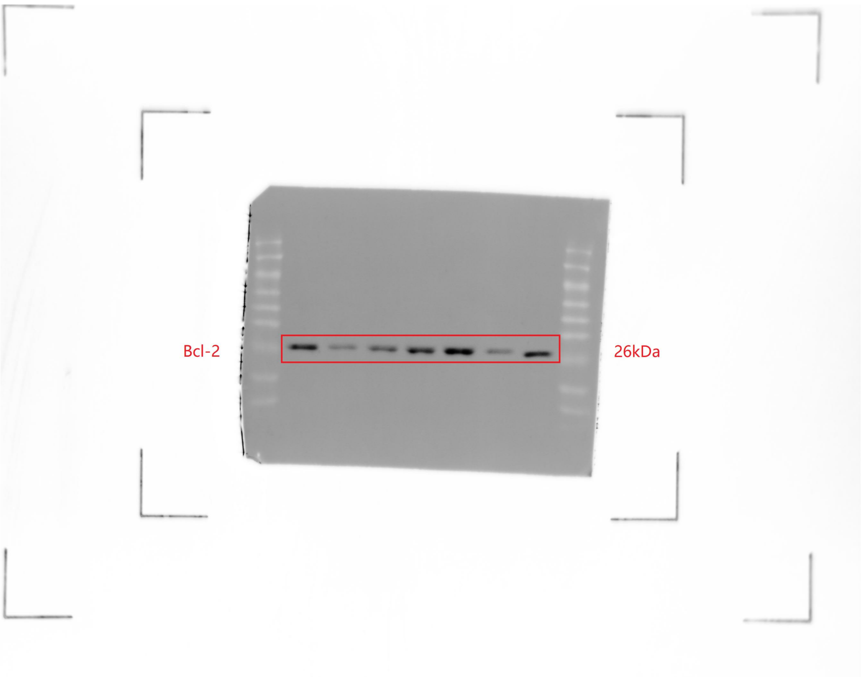
Bcl-2
26kDa

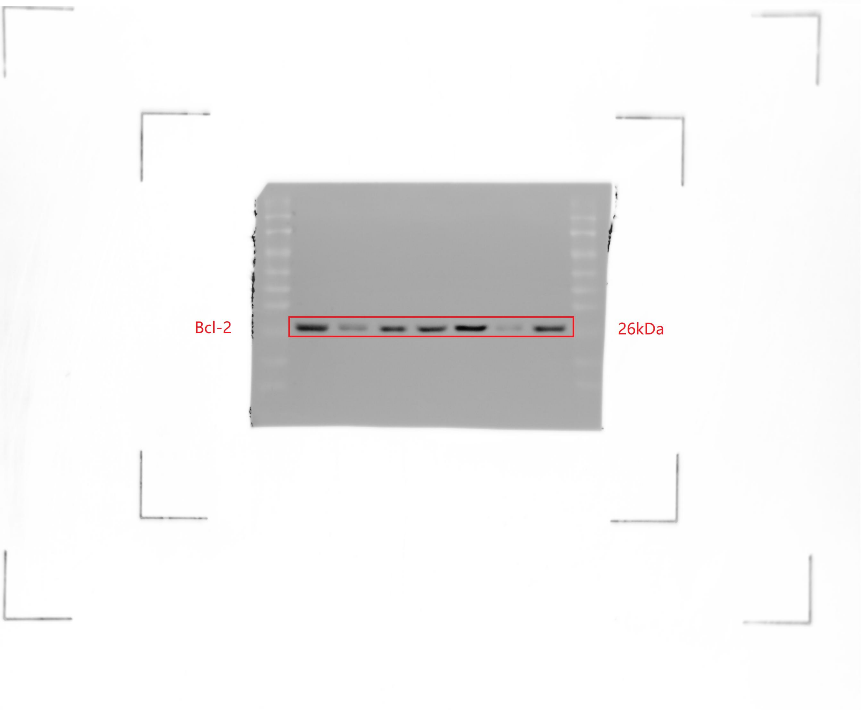
Bcl-2
26kDa

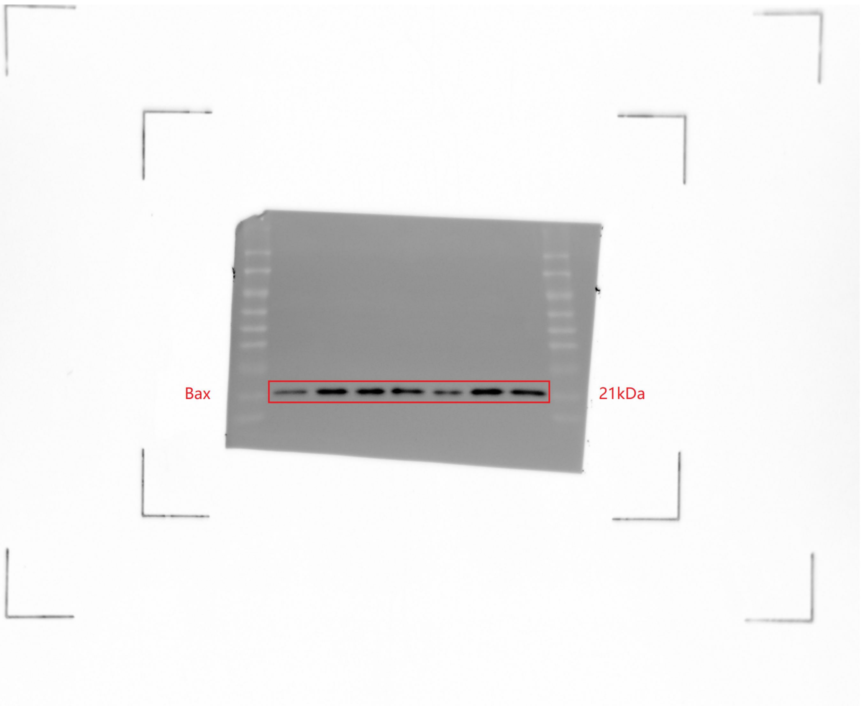
Bax
21kDa

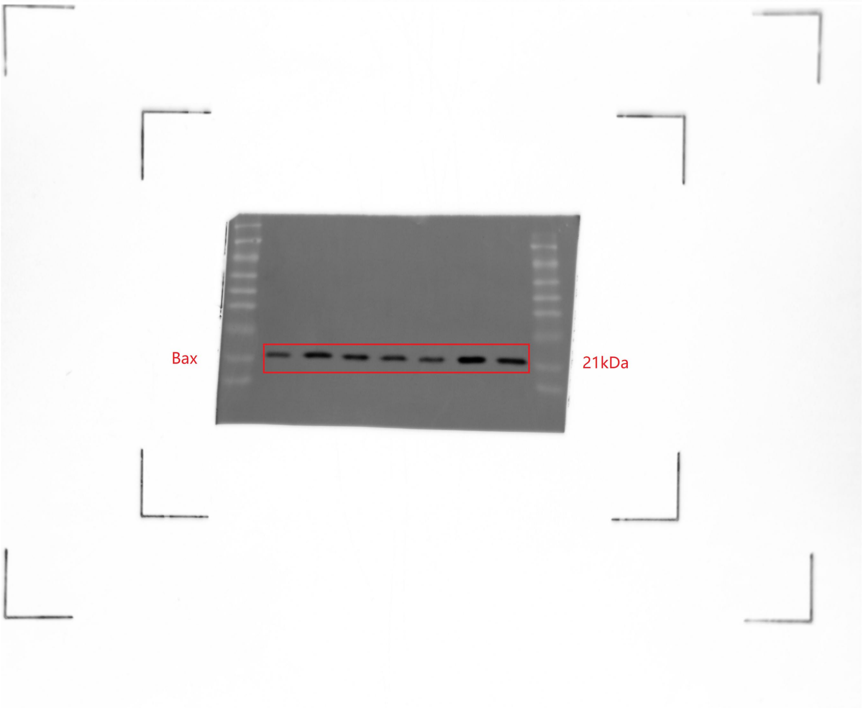
Bax
21kDa

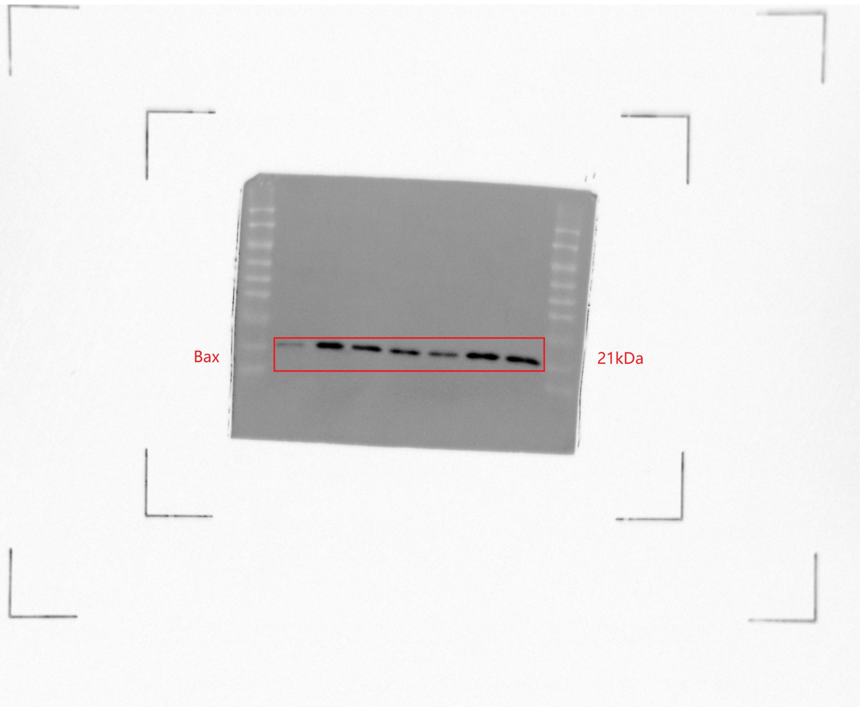
Bax
21kDa

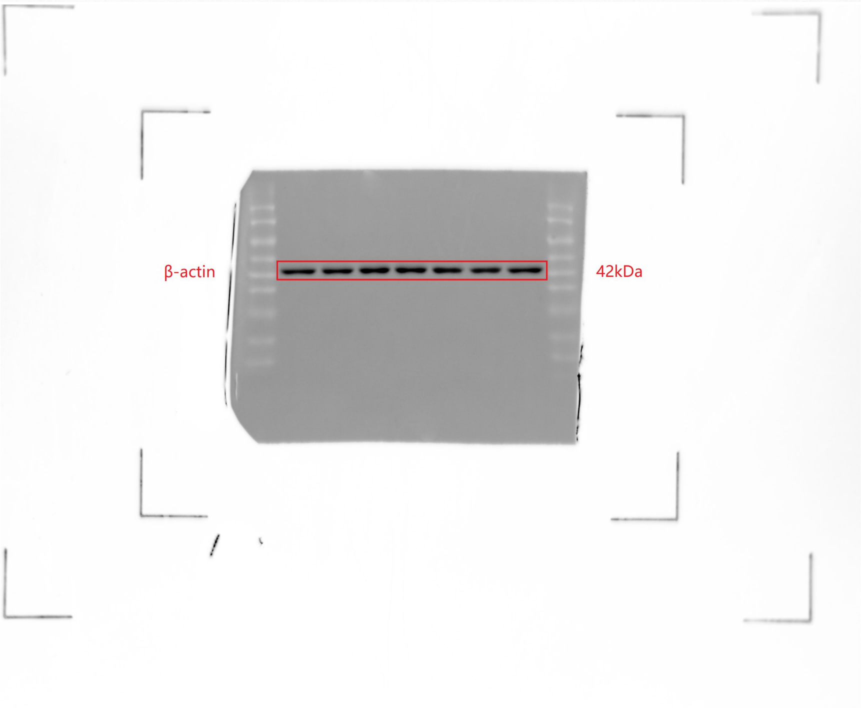
β-actin
42kDa

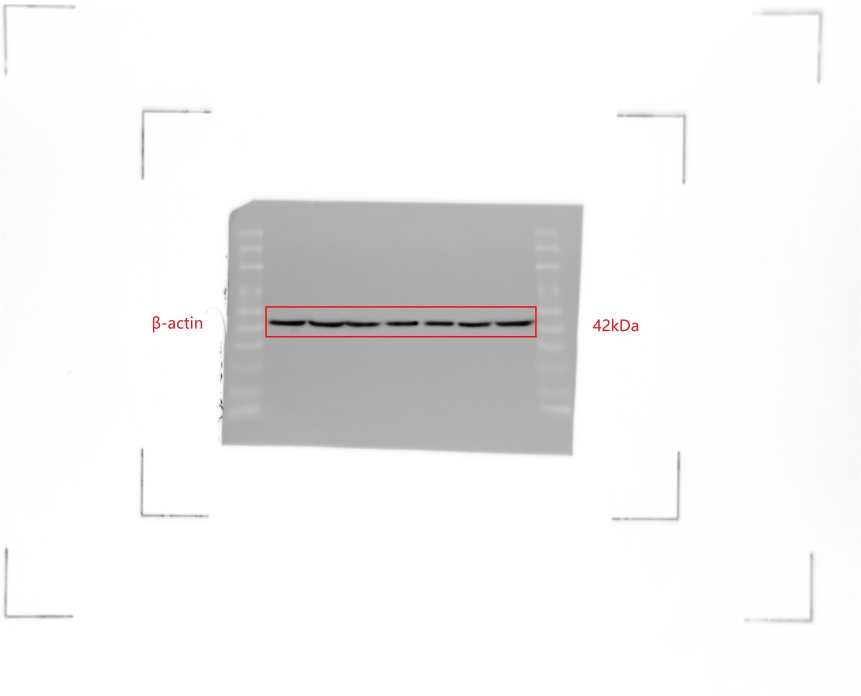
β-actin
42kDa

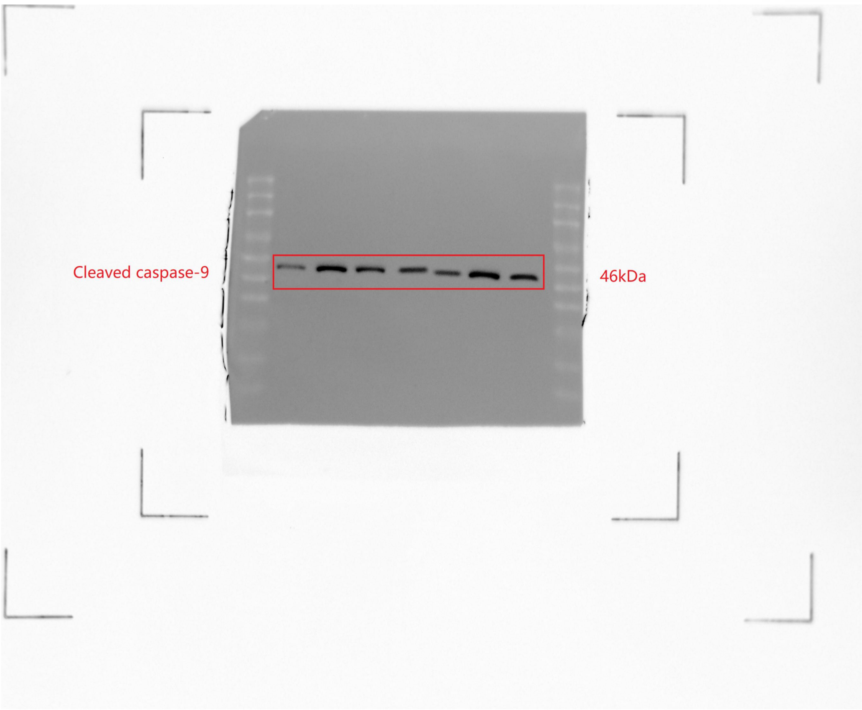
Cleaved caspase-9
46kDa

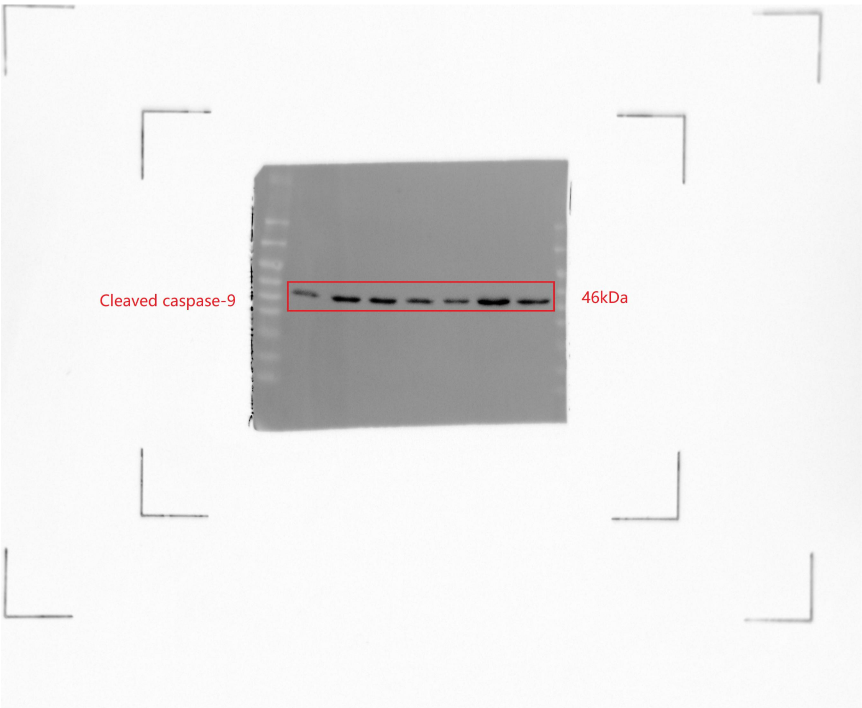
Cleaved caspase-9
46kDa

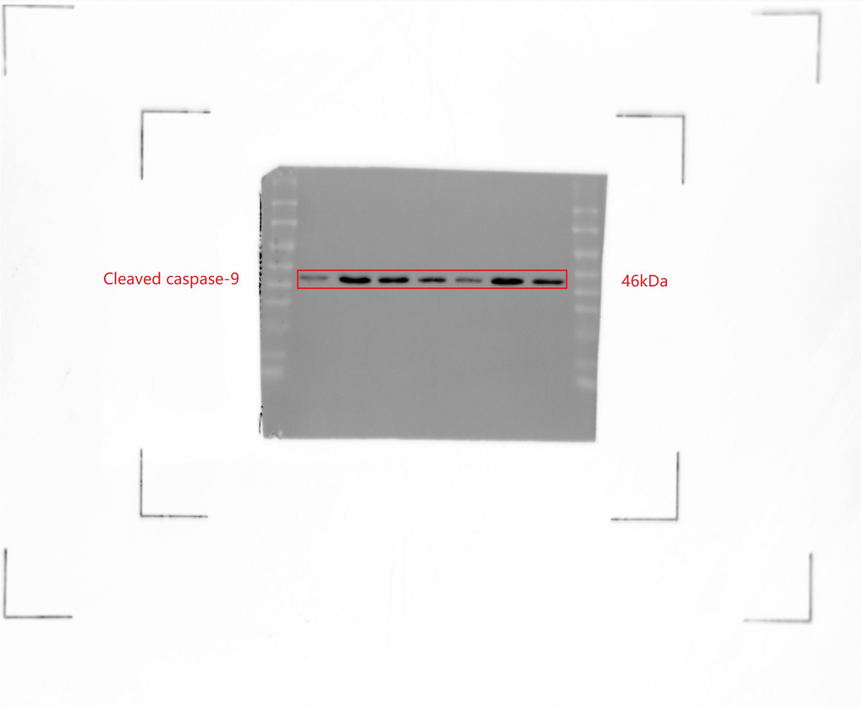
Cleaved caspase-9
46kDa

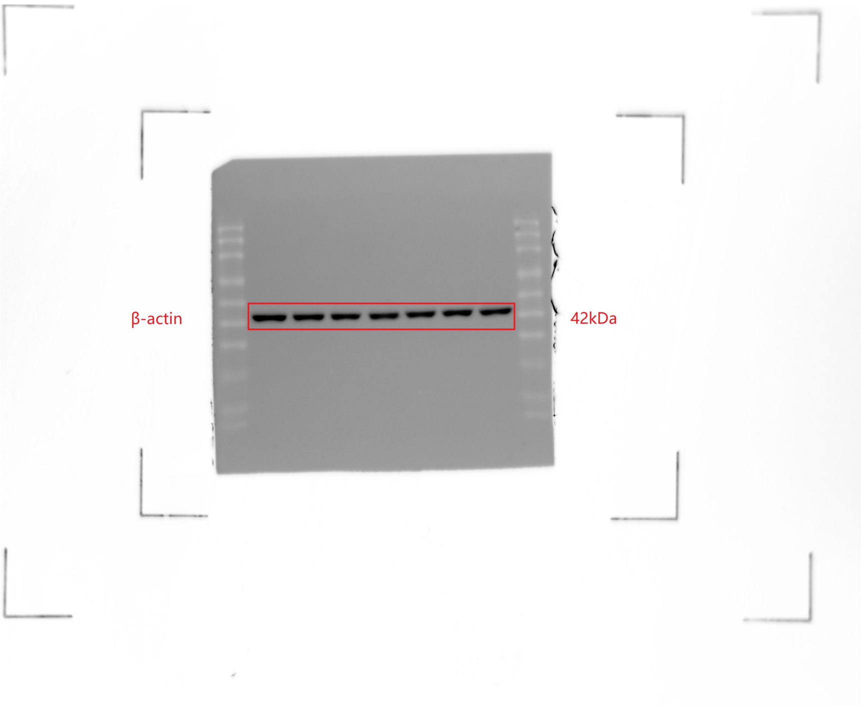
β-actin
42kDa
