# Supplementary material for: High-temperature requirement serine protease A2 inhibitor UCF-101 ameliorates damaged neurons in traumatic brain-injured rats by the AMPK/NF-κB pathway
Source: Open Life Sci. 2025 Mar 6;20(1):20220971. doi: 10.1515/biol-2022-0971 (PMC11889502; doi:10.1515/biol-2022-0971)
Supplement: Supplementary material-2 [file biol-2022-0971-sm2.pdf]

**Resolution Paper of Laboratory Animal Management and Welfare Ethical Review Committee**

| | |
|---|---|
| Number of resolution | ZJEY-20230615-04 |
| Name of experiment | High-Temperature Requirement Serine Protease A2 Inhibitor UCF-101 Attenuates Neuronal Damage Inflammation and Effects on |
| Number of acception | 202306-1304 |
| Time of applicaiton | 2023.06.13 |
| Whether through the first trial | ☑ Adoption ☐ No adoption |
| Time of review | 2023-06-15 |
| Conclusive opinion | ☑ Meet the requirements of Animal Care and Welfare Committee<br>☐ Need to adjust experimental scheme |
| Review of resolution | ☑ The research can be performed<br>☐ After adjusting experimental protocol, the research can be performed |
| Signature of detector of Animal Care and Welfare Committee | |

Official seal:
